# Rho-associated kinase and zipper-interacting protein kinase, but not myosin light chain kinase, are involved in the regulation of myosin phosphorylation in serum-stimulated human arterial smooth muscle cells

**DOI:** 10.1371/journal.pone.0226406

**Published:** 2019-12-13

**Authors:** Jing-Ti Deng, Sabreena Bhaidani, Cindy Sutherland, Justin A. MacDonald, Michael P. Walsh

**Affiliations:** Department of Biochemistry & Molecular Biology, Cumming School of Medicine, University of Calgary, Calgary, Alberta, Canada; Duke University School of Medicine, UNITED STATES

## Abstract

Myosin regulatory light chain (LC_20_) phosphorylation plays an important role in vascular smooth muscle contraction and cell migration. Ca^2+^/calmodulin-dependent myosin light chain kinase (MLCK) phosphorylates LC_20_ (its only known substrate) exclusively at S19. Rho-associated kinase (ROCK) and zipper-interacting protein kinase (ZIPK) have been implicated in the regulation of LC_20_ phosphorylation via direct phosphorylation of LC_20_ at T18 and S19 and indirectly via phosphorylation of MYPT1 (the myosin targeting subunit of myosin light chain phosphatase, MLCP) and Par-4 (prostate-apoptosis response-4). Phosphorylation of MYPT1 at T696 and T853 inhibits MLCP activity whereas phosphorylation of Par-4 at T163 disrupts its interaction with MYPT1, exposing the sites of phosphorylation in MYPT1 and leading to MLCP inhibition. To evaluate the roles of MLCK, ROCK and ZIPK in these phosphorylation events, we investigated the time courses of phosphorylation of LC_20_, MYPT1 and Par-4 in serum-stimulated human vascular smooth muscle cells (from coronary and umbilical arteries), and examined the effects of siRNA-mediated MLCK, ROCK and ZIPK knockdown and pharmacological inhibition on these phosphorylation events. Serum stimulation induced rapid phosphorylation of LC_20_ at T18 and S19, MYPT1 at T696 and T853, and Par-4 at T163, peaking within 30–120 s. MLCK knockdown or inhibition, or Ca^2+^ chelation with EGTA, had no effect on serum-induced LC_20_ phosphorylation. ROCK knockdown decreased the levels of phosphorylation of LC_20_ at T18 and S19, of MYPT1 at T696 and T853, and of Par-4 at T163, whereas ZIPK knockdown decreased LC_20_ diphosphorylation, but *increased* phosphorylation of MYPT1 at T696 and T853 and of Par-4 at T163. ROCK inhibition with GSK429286A reduced serum-induced phosphorylation of LC_20_ at T18 and S19, MYPT1 at T853 and Par-4 at T163, while ZIPK inhibition by HS38 reduced only LC_20_ diphosphorylation. We also demonstrated that serum stimulation induced phosphorylation (activation) of ZIPK, which was inhibited by ROCK and ZIPK down-regulation and inhibition. Finally, basal phosphorylation of LC_20_ in the absence of serum stimulation was unaffected by MLCK, ROCK or ZIPK knockdown or inhibition. We conclude that: (i) serum stimulation of cultured human arterial smooth muscle cells results in rapid phosphorylation of LC_20_, MYPT1, Par-4 and ZIPK, in contrast to the slower phosphorylation of kinases and other proteins involved in other signaling pathways (Akt, ERK1/2, p38 MAPK and HSP27), (ii) ROCK and ZIPK, but not MLCK, are involved in serum-induced phosphorylation of LC_20_, (iii) ROCK, but not ZIPK, directly phosphorylates MYPT1 at T853 and Par-4 at T163 in response to serum stimulation, (iv) ZIPK phosphorylation is enhanced by serum stimulation and involves phosphorylation by ROCK and autophosphorylation, and (v) basal phosphorylation of LC_20_ under serum-free conditions is not attributable to MLCK, ROCK or ZIPK.

## Introduction

Phosphorylation at S19 of the 20-kDa regulatory light chains of myosin II (LC_20_), catalyzed by myosin light chain kinase (MLCK), is the trigger for smooth muscle contraction [1–4]. Smooth muscle MLCK is capable of phosphorylating T18 of LC_20_ in addition to S19 [5,6]; however, this has only been demonstrated *in vitro* at high kinase concentrations (a high MLCK: myosin II ratio), and abundant evidence indicates that it does not occur *in situ* [7,8]. In some instances, however, LC_20_ is phosphorylated at both T18 and S19 in vascular smooth muscle tissue. For example, in renal afferent arterioles of the rat, the pathophysiological stimulus endothelin-1 induces diphosphorylation of LC_20_ at T18 and S19, whereas the physiological stimulus angiotensin II induces exclusively monophosphorylation at S19 [9]. Diphosphorylation is associated with a decrease in the rate of dephosphorylation of LC_20_ by myosin light chain phosphatase (MLCP) and, therefore, prolongation of the contractile response [10]. LC_20_ diphosphorylation has also been observed frequently in cultured smooth muscle and endothelial cells [11–13]. The observation that LC_20_ diphosphorylation occurs in response to treatment with membrane-permeant phosphatase inhibitors indicates that blocking phosphatase activity unmasks basal activity of endogenous kinase(s) that phosphorylate LC_20_ at T18 and S19 [14]. These findings raise the question: which kinase(s) is responsible for phosphorylation of LC_20_ at T18 and S19? Candidate kinases include Rho-associated coiled-coil kinase (ROCK) [15], zipper-interacting protein kinase (ZIPK) [16–20], integrin-linked kinase (ILK) [21,22] and citron kinase (a Rho-dependent kinase related to ROCK) [23]. In this study, we followed the time courses of phosphorylation of LC_20_ and of proteins implicated in the regulation of myosin light chain phosphorylation, the myosin targeting subunit of MLCP (MYPT1), prostate-apoptosis response-4 (Par-4) and ZIPK itself, in serum-stimulated human cultured vascular smooth muscle cells from coronary artery and umbilical artery. We then investigated the effects of MLCK, ROCK and ZIPK knockdown and pharmacological inhibition on the serum-induced phosphorylation of LC_20_, MYPT1, Par-4 and ZIPK.

## Materials and methods

### Materials

All chemicals were reagent grade unless indicated otherwise. H1152 ((*S*)-(+)-2-methyl-1-[(4-methyl-5-isoquinolinyl)sulfonyl]-hexahydro-1*H*-1,4-diazepine dihydrochloride), a selective ROCK inhibitor [24] purchased from Calbiochem (catalog number 555550), was used at 200 nM; it also inhibits, but with lower potency, PRK2 and other members of the AGC subfamily of protein kinases such as RSK1, RSK2, PKA, MSK1 [25] and LRRK2 [26]. GSK429286A (*N*-(6-fluoro-1H-indazol-5-yl)-2-methyl-6-oxo-4-(4-(trifluoromethyl)phenyl)-1,4,5,6-tetrahydropyridine-3-carboxamide), a more selective ROCK inhibitor that does not inhibit LRRK2, was used at 1 μM [26] and was purchased from Selleckchem (Houston, TX; cat. no. S1474). HS38 (2-((1-(3-chlorophenyl)-4-oxo-4,5-dihydro-1H-pyrazolo[3,4-*d*]-pyrimidin-6-yl)thio)propanamide), a ZIPK inhibitor [27,28], was generously provided by Dr. Tim Haystead (Duke University). HS38 has no effect on ROCK activity [29], unlike other commercially available compounds such as TC-DAPK6 [29,30]; HS38 does, however, inhibit PIM kinases and IRAK4 [27]. Wortmannin, purchased from Calbiochem (cat. no. W1628), was used at a concentration of 1 μM, at which it is a highly selective inhibitor of PI 3-kinase and MLCK [31]. ML7 (1-(5-iodonaphthalene-1-sulfonyl)-1H-hexahydro-1,4-diazepine hydrochloride), a selective MLCK inhibitor purchased from Calbiochem (cat. no. I2764), was used at 20 μM [32].

### Human arterial smooth muscle cell cultures

Human coronary artery smooth muscle cells (CASMC: CC-2583) and human umbilical artery smooth muscle cells (UASMC: CC-2579), purchased from Lonza (Basel, Switzerland), were cultured in smooth muscle growth medium (Lonza SmGM-2: CC-3182) containing 5% fetal bovine serum (FBS), growth factors (hEGF, insulin and hFGF-β), gentamicin and amphotericin-B at 37 °C in a humidified incubator with 5% CO_2_. These cells maintain their morphological and phenotypic characteristics for up to 10 passages and were, therefore, used for experiments within 10 passages. Cells were plated at a density of 2 X 10^5^ cells/ml into 4-well plates (Nunc, Thermo Scientific, Waltham, MA) with smooth muscle growth medium containing 5% FBS. Prior to treatment, cells were grown to 70% confluence and starved in FBS-free medium overnight. Cells were then stimulated with 5% FBS for times indicated in the figures (typically 0, 15 s, 30 s, 1 min, 2 min, 5 min, 30 min, 1 h and 2 h) to observe their various responses to FBS. The treated cells were lysed immediately in Laemmli sample buffer and heated at 90 °C for 5 min. To investigate the effects of kinase inhibitors, cells were starved in FBS-free medium for 8 h to induce cell quiescence and then incubated with various inhibitors for ≥ 45 min prior to stimulation with 5% FBS for 2 min and then lysed in Laemmli sample buffer.

### siRNA-mediated knockdown

CASMC and UASMC were transfected with human ROCK1, ZIPK or MLCK siRNAs or negative control siRNA (see below) using HiPerFect Reagent (Qiagen, Hilden, Germany) according to the manufacturer’s protocol, as previously described in detail [33]. Human ROCK1 siRNA, purchased from Santa Cruz Biotechnology (Santa Cruz, CA; cat. no. sc-29473), consists of three target-specific siRNAs (19–25 nucleotides). Human ZIPK siRNA, purchased from Ambion/Thermo Fisher Scientific (Waltham, MA; part number 4390824, siRNA ID #s559, Lot #ASO0UMMU), has the following sequence:

sense: 5'-GAGGAGUACUUCAGCAACAtt-3'
antisense: 5'-UGUUGCUGAAGUACUCCUCgt-3'.

Human MLCK siRNA, purchased from Ambion/Thermo Fisher Scientific (part number 4392420, siRNA ID#s9193, Lot #ASO0T348), has the following sequence:

sense: 5'-GCCUCAUGUAAAACCCUAUtt-3'
anti-sense: 5'-AUAGGGUUUUACAUGAGGCtt-3'.

Negative control siRNA (Ambion negative control #2, part #AM4613) contains sequences that do not target any gene product.

### Western blotting

Cells were lysed in Laemmli sample buffer (65 mM Tris-HCl, pH 6.8, 10% glycerol, 3% SDS, 1% β-mercaptoethanol, 0.04% bromophenol blue) following treatments indicated in the Results section, heated at 90 °C for 5 min and resolved by SDS-PAGE (11% acrylamide). Proteins were transferred to nitrocellulose membranes in 25 mM Tris, 192 mM glycine, 20% methanol overnight at 30 V and 4 °C. Membranes were blocked with 5% non-fat dry milk in 25 mM Tris-HCl, pH 7.4, 150 mM NaCl, 0.05% Tween-20 (TBST) for 1 h at 20 °C. Membranes were incubated for 2 h at 20 °C with primary antibody in 1% milk in TBST, washed, incubated with horseradish peroxidase (HRP)-conjugated goat polyclonal anti-rabbit IgG (1:100,000 dilution; EMD Millipore (Etobicoke, Ontario, Canada); cat. no. AP132P) or donkey anti-goat IgG-HRP (1:100,000 dilution; Santa Cruz Biotechnology; cat. no. sc-2020) in 1% milk in TBST and washed 3 times with TBST. Blots were developed with Supersignal West Femto enhanced chemiluminescence substrate (Thermo Scientific). Emitted light was detected and quantified with a chemiluminescence imaging analyzer (LAS3000mini; Fujifilm, Tokyo, Japan) and images were analyzed with MultiGauge version 3.0 software (Fujifilm). Protein levels were expressed relative to a standard (GAPDH, SM22α or α-actin) as indicated in the text. The following primary antibodies were used in this study: rabbit monoclonal anti-ZIPK (1:1,000 dilution; Epitomics, Burlingame, CA; cat. no. 2568–1), rabbit polyclonal anti-ROCK1 (1:1,000 dilution; Epitomics; cat. no. S2711), rabbit polyclonal anti-ROCK2 (1:2,000 dilution; AbFrontier, Seoul, South Korea; cat. no. LF-PA0049), rabbit polyclonal anti-pT18/pS19-myosin light chain 2 (1:500 dilution; Cell Signaling; cat. no. 3674), rabbit anti-GAPDH (1:5,000 dilution; Santa Cruz Biotechnology; cat. no. sc-32233), goat polyclonal anti-SM22α (1:2,500 dilution; Novus Biologicals, Centennial, CO; cat. no. NB600-507), rabbit polyclonal anti-LC_20_ (1:2,000 dilution; Rockland Immunochemicals, Limerick, PA; cat. no. 600-401-938), rabbit polyclonal anti-pT853-MYPT1 (1:1,000 dilution; EMD Millipore; cat. no. 36–003), rabbit polyclonal anti-pT696-MYPT1 (1:1,000 dilution; EMD Millipore; cat. no. ABS45), rabbit polyclonal anti-pT163-Par4 (1:1,000 dilution; Cell Signaling; cat. no. 2329), rabbit monoclonal anti-pS473-Akt (193H12) (1:1,000 dilution; Cell Signaling; cat. no. 4058); rabbit polyclonal anti-pT180/pY182-p38 MAP kinase (1:1,000 dilution; Cell Signaling; cat. no. 9211), rabbit monoclonal anti-pT202/Y204-ERK1/pT185/pY187-ERK2 (1:1,000 dilution; Cell Signaling; cat. no. 4370), rabbit anti-pS82-HSP27 (1:1,000 dilution; Cell Signaling; cat. no. 2401), and rabbit polyclonal anti-α-actin (1:1,000 dilution; Cytoskeleton, Inc.; cat. no. AAN01). Polyclonal antibodies to purified, full-length chicken gizzard MLCK were raised in rabbits [34].

### Phosphate affinity SDS-PAGE (Phos-tag SDS-PAGE)

The phosphorylation of LC_20_ and ZIPK was analyzed by phosphate-binding tag SDS-PAGE [35]. For LC_20_ phosphorylation analysis, samples were resolved in SDS gels containing 12% acrylamide, 40 μM Phos-tag reagent and 0.1 mM MnCl_2_ at 20 mA/gel. Separated proteins were transferred to PVDF membranes (Roche Applied Science, Penzberg, Germany) overnight at 28 V and 4 °C in 25 mM Tris-HCl, pH 7.5, 192 mM glycine, 10% (v/v) methanol. Proteins were fixed on the membrane by treatment with 0.5% glutaraldehyde in phosphate-buffered saline (137 mM NaCl, 2.68 mM KCl, 10 mM Na_2_HPO_4_, 1.76 mM KH_2_PO_4_) for 30 min. For ZIPK phosphorylation analysis, samples were separated in SDS gels containing 6.5% acrylamide, 55 μM Phos-tag reagent and 0.1 mM MnCl_2_ (or 1–2 mM EDTA) at 20 mA/gel. Separated proteins were transferred to nitrocellulose membranes in 25 mM Tris-HCl, pH 7.5, 192 mM glycine, 20% (v/v) methanol at 28 V for 18 h at 4 °C. Membranes were then blocked with 5% non-fat dry milk in TBST for 1 h at 20 °C, washed with water and TBST and incubated for 2 h with primary antibody (anti-ZIPK) in Can Get Signal (Immunoreaction Enhancer Solution 1; Toyobo Life Science Department, Osaka, Japan) prior to further washing in water and TBST and incubation for 1 h with secondary antibody in 1% (w/v) non-fat dry milk in TBST. Immunoreactive bands were detected with SuperSignal West Femto Maximum Sensitivity Substrate (Thermo Fisher Scientific).

### Data analysis

Values are presented as mean ± SEM, with *n* indicating the number of independent experiments performed. Statistically significant differences were determined by Student’s unpaired *t*-test or Dunnett’s *post hoc* test for multiple comparisons as indicated in the text and figure legends. Statistically significant differences were indicated by *p* < 0.05.

## Results

### Serum-induced phosphorylation of LC_20_

**Fig 1:**
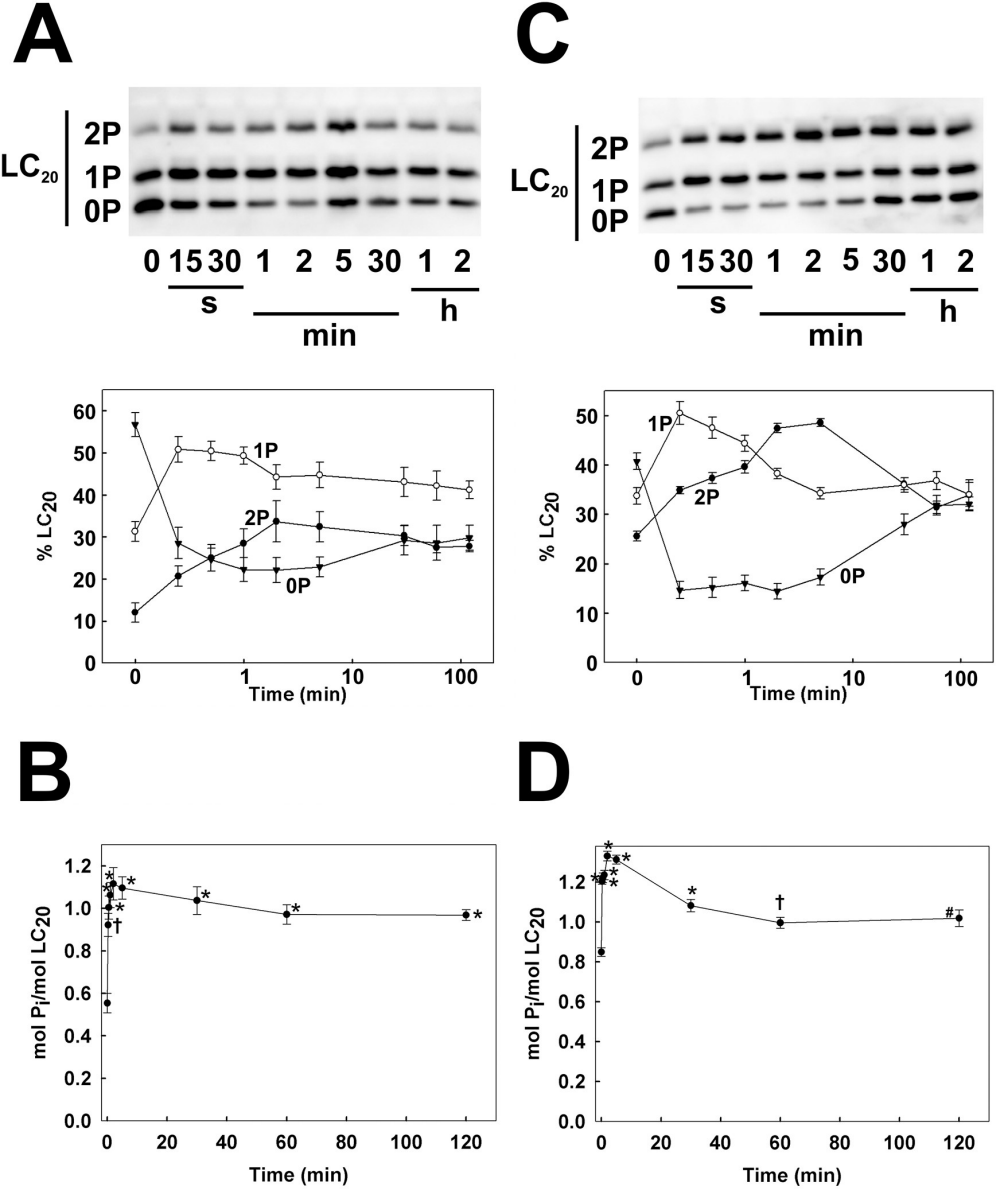
Time courses of serum-induced LC_20_ phosphorylation in human arterial smooth muscle cells. Human coronary (A, B) and umbilical arterial smooth muscle cells (C, D) were seeded in 4-well plates, serum starved overnight and stimulated with 5% FBS at time zero. Cells were lysed in Laemmli sample buffer at the indicated times of serum stimulation and subjected to Phos-tag SDS-PAGE and western blotting with anti-LC_20_ to detect unphosphorylated (0P), mono- (1P) and diphosphorylated LC_20_ (2P). Representative western blots are shown in panels A and C above cumulative quantitative data for each of the LC_20_ bands. Panels B and D show the time courses of total phosphorylation stoichiometry, calculated as follows: mol P_i_/mol LC_20_ = (1P + 2x2P) / (0P + 1P + 2P). Values indicate the mean ± SEM (*n* = 7 for panels A and B; *n* = 6 for panels C and D). Significant differences from the value at time zero are indicated: *p < 0.0001, ^†^*p* = 0.0001 (B), *p < 0.0001, ^†^*p* = 0.0023, ^#^*p* = 0.0004 (D) (Dunnett’s *post hoc* test).

**Fig 2:**
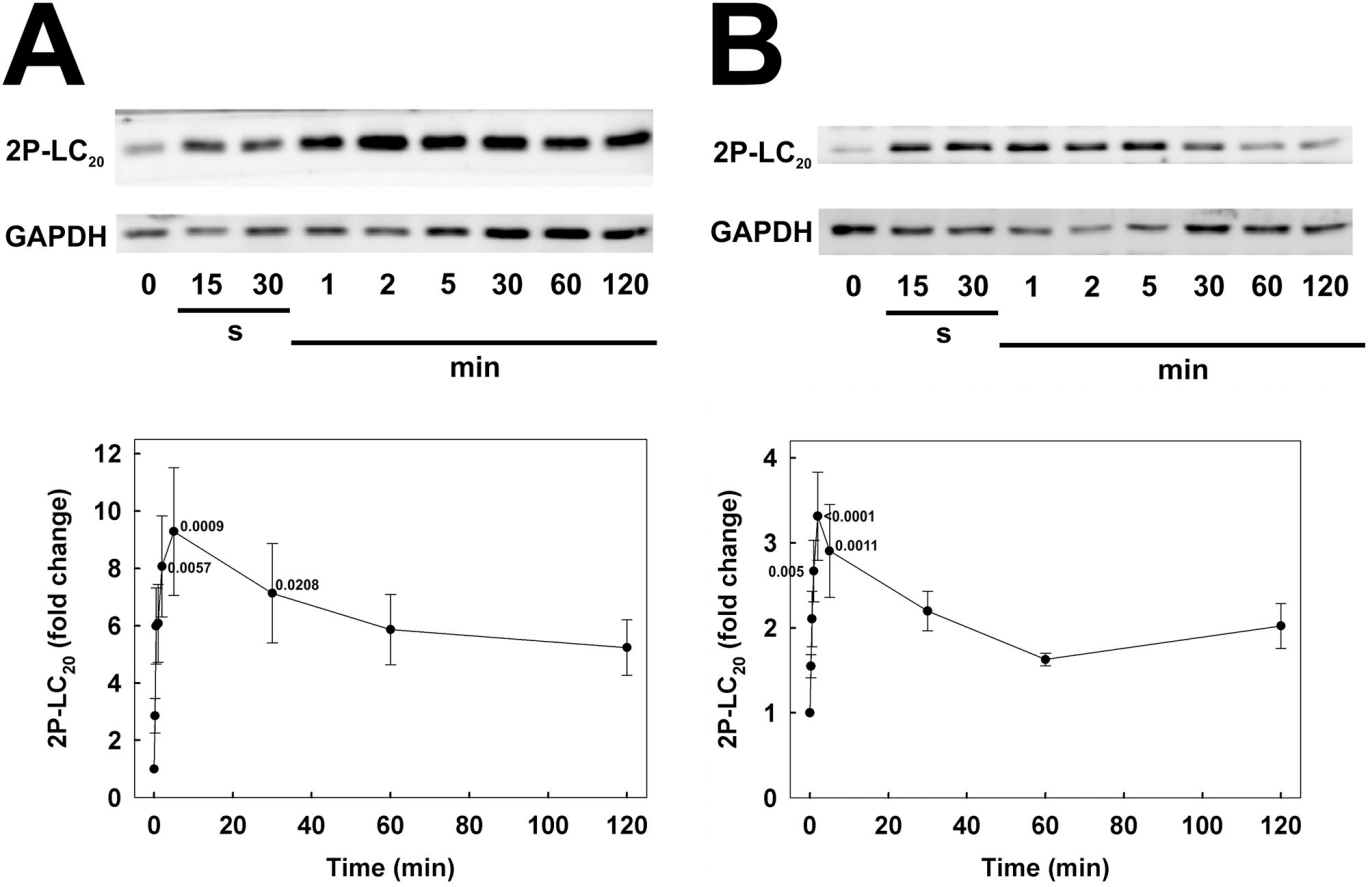
Confirmation of serum-induced LC_20_ diphosphorylation at T18 and S19. Human coronary (A) and umbilical arterial smooth muscle cells (B) were treated with serum at time zero, as described in the legend to Fig 1, and diphosphorylation of LC_20_ at T18 and S19 was assessed by regular SDS-PAGE and western blotting with an antibody that specifically recognizes LC_20_ only when phosphorylated at both T18 and S19. Representative western blots are shown in each panel above cumulative quantitative data depicting the time-dependent changes in LC_20_ diphosphorylation expressed as fold change relative to the value at time zero (prior to addition of serum) after normalization of loading levels using GAPDH. Values indicate the mean ± SEM (*n* = 6). Significant differences from the value at time zero are indicated with their respective *p* values (Dunnett’s *post hoc* test).

Analysis of LC_20_ phosphorylation in serum-starved human coronary and umbilical arterial smooth muscle cells by Phos-tag SDS-PAGE, which separates unphosphorylated, mono- and diphosphorylated LC_20_ species [10], revealed a significant basal level of phosphorylation: 0.55 ± 0.05 mol P_i_/mol LC_20_ (*n* = 7) in CASMC and 0.85 ± 0.02 mol P_i_/mol LC_20_ (*n* = 6) in UASMC (time zero samples in Fig 1B and 1D, respectively). Most of the basal phosphorylation under these conditions was at a single site, although diphosphorylated LC_20_ was also detected (time zero samples in Fig 1A and 1C). Serum stimulation induced a rapid increase in phosphorylation to a peak of 1.12 ± 0.08 mol P_i_/mol LC_20_ (*n* = 7) in CASMC (Fig 1A and 1B) and 1.33 ± 0.02 mol P_i_/mol LC_20_ (*n* = 6) in UASMC (Fig 1C and 1D) at 2 min after serum addition. In the case of CASMC, this level of LC_20_ phosphorylation was sustained for a prolonged period of time; the value after 2 h serum stimulation was 0.97 ± 0.03 mol P_i_/mol LC_20_ (*n* = 7), not significantly different from the value at 2 min (*p* = 0.09; Student’s unpaired *t*-test) (Fig 1B), whereas it declined significantly over this period in UASMC to 1.02 ± 0.04 mol P_i_/mol LC_20_ (*n* = 6), significantly lower than the value at 2 min (*p* < 0.001; Student’s unpaired *t*-test) (Fig 1D). Analysis of LC_20_ diphosphorylation using phosphospecific antibodies that recognize LC_20_ only when phosphorylated at both T18 and S19 confirmed a low level of basal diphosphorylation under serum-free conditions, which increased rapidly in both CASMC (Fig 2A) and UASMC (Fig 2B) in response to serum stimulation and then significantly declined in UASMC (*p* < 0.02 *versus* peak value; Student’s unpaired *t*-test) to steady-state levels significantly above resting values (Fig 2B); this is also demonstrated by the diphosphorylated band in the Phos-tag gels in Fig 1A and 1C. There was no significant decline in diphosphorylation of LC_20_ over the 2-h incubation period in CASMC (Fig 2A; *p* = 0.13 *versus* peak value; Student’s unpaired *t*-test).

### Serum-induced phosphorylation of MYPT1

**Fig 3:**
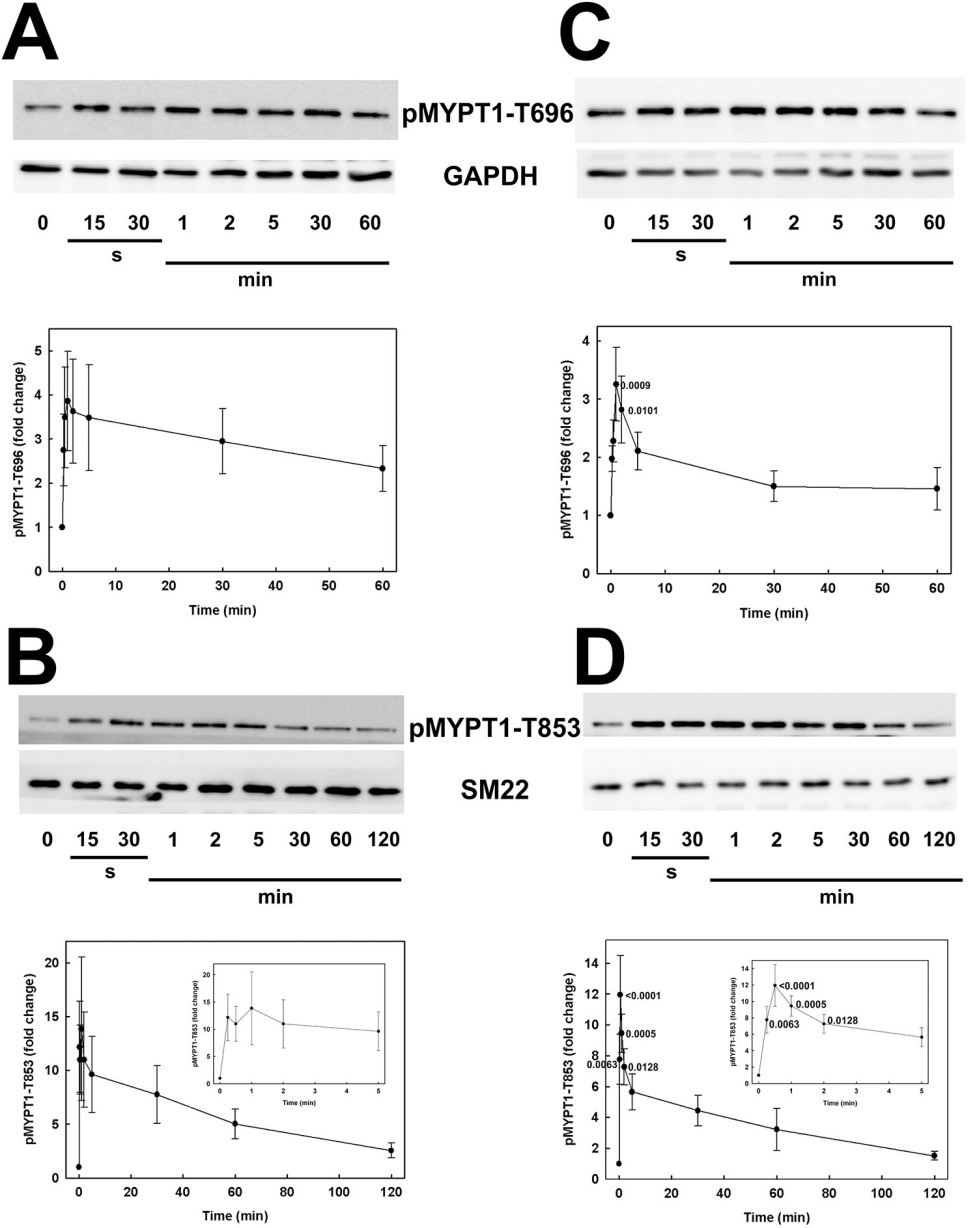
Time courses of serum-induced MYPT1 phosphorylation at T696 and T853 in human arterial smooth muscle cells. Human coronary (A, B) and umbilical arterial smooth muscle cells (C, D) were treated with serum at time zero, as described in the legend to Fig 1. Cells were lysed in Laemmli sample buffer at the indicated times and subjected to SDS-PAGE and western blotting with anti-pT696-MYPT1 (A, C) or anti-pT853-MYPT1 (B, D). Representative western blots are shown in each panel above cumulative quantitative data. Phosphorylated MYPT1 (pMYPT1) signals were normalized to GAPDH (A, C) or SM22 (B, D) and expressed relative to the pMYPT1: GAPDH or pMYPT1: SM22 ratio at time zero. Values indicate the mean ± SEM (*n* = 7 in A; *n* = 8 in B; *n* = 6 in C and D). Significant differences from the value at time zero are indicated with their respective *p* values (Dunnett’s *post hoc* test). Insets in B and D show the first 5 min of the time courses for greater clarity.

Analysis of MYPT1 phosphorylation at T696 and T853 by western blotting with phosphospecific antibodies revealed a low basal level of phosphorylation at both sites under serum-free conditions in CASMC (Fig 3A and 3B) and UASMC (Fig 3C and 3D). Serum stimulation induced a rapid increase in phosphorylation at both sites in both cell types, which peaked at 1–2 min and declined steadily with time, in most cases returning to basal levels within 2 h of exposure to serum.

### Serum-induced phosphorylation of Par-4

**Fig 4:**
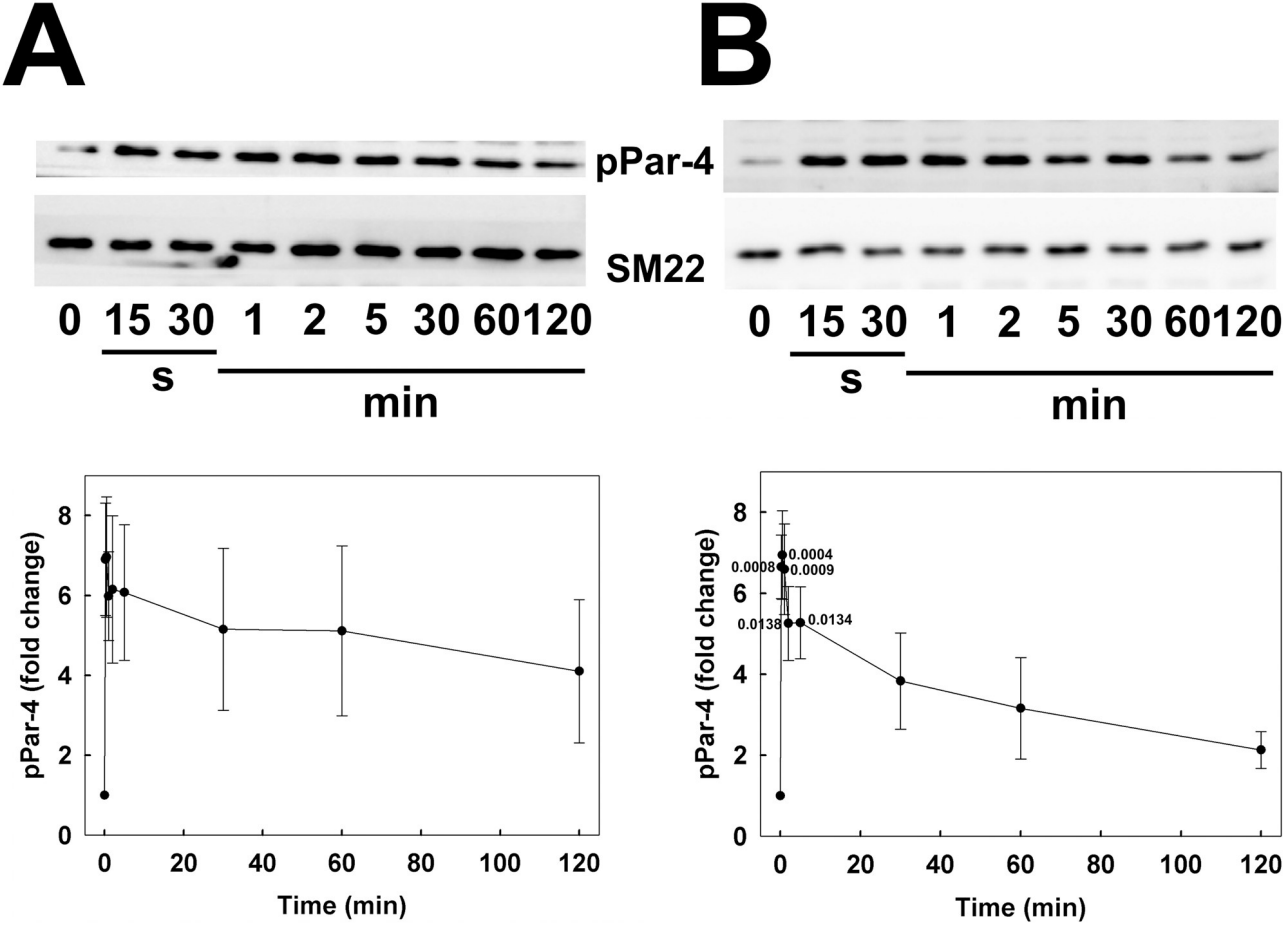
Time courses of serum-induced Par-4 phosphorylation at T163 in human arterial smooth muscle cells. Human coronary (A) and umbilical arterial smooth muscle cells (B) were treated with serum at time zero, as described in the legend to Fig 1. Cells were lysed in Laemmli sample buffer at the indicated times and subjected to SDS-PAGE and western blotting with anti-pT163-Par-4. Representative western blots are shown in each panel above cumulative quantitative data. Phospho-Par-4 signals were normalized to SM22 and expressed relative to the pPar-4: SM22 ratio at time zero. Values indicate the mean ± SEM (*n* = 6). Significant differences from the value at time zero are indicated with their respective *p* values (Dunnett’s *post hoc* test). The SM22 controls in panels A and B are the same as those in Fig 3B and 3D, respectively, since all three proteins (pMYPT1-T853, pPar-4 and SM22) were analysed on the same gel/blot.

Analysis of Par-4 phosphorylation at T163 by western blotting with phosphospecific antibodies revealed a low basal level of phosphorylation under serum-free conditions in CASMC (Fig 4A) and UASMC (Fig 4B). Serum stimulation induced a rapid increase in phosphorylation in both cell types, which peaked at 30 s and declined towards basal levels over the 2-h incubation period.

### Serum-induced phosphorylation of Akt

We also investigated the time courses of serum-induced phosphorylation events distinct from the signaling pathways involved in LC_20_ phosphorylation, specifically the kinases Akt/PKB, ERK1/2 and p38 MAPK as well as the kinase substrate, heat shock protein HSP27. PI3K/Akt signaling in VSMCs is involved in the etiology of atherosclerosis [36,37]. Phosphorylation at T308 and S473 of Akt/PKB is required for full activation [38]. Analysis of Akt phosphorylation at S473 by western blotting with phosphospecific antibodies revealed a low basal level of phosphorylation under serum-free conditions in CASMC (S1A Fig) and UASMC (S1B Fig). Serum stimulation induced a slower rate of increase in phosphorylation in both cell types compared to the phosphorylation events described above, peaked at 5–30 min and remained elevated over the 2-h incubation period.

### Serum-induced phosphorylation of ERK1/2

The ERK MAPKs, ERK1/p44 and ERK2/p42, are activated by a wide variety of growth factors and mitogens via phosphorylation at the T and Y residues (T202 and Y204 in ERK1 and T185 and Y187 in ERK2) within the activation loop TEY sequence [39]. ERK1 and ERK2 have been implicated in cell migration [40]. Analysis of ERK1 and ERK2 phosphorylation by western blotting with phosphospecific antibodies revealed a low basal level of phosphorylation under serum-free conditions in CASMC (S2 Fig). Serum stimulation induced a relatively slow increase in phosphorylation of both 44-kDa ERK1 (S2B Fig) and 42-kDa ERK2 (S2C Fig), which peaked at 5 min and remained elevated over the 1-h incubation period. Similar results were observed in UASMC, in which case total ERK phosphorylation was quantified due to the difficulty of achieving clear separation of the two isoforms (S3 Fig).

### Serum-induced phosphorylation of p38 MAPK

p38 MAPK has also been implicated in cell migration through activation by several growth factors, cytokines and chemotactic substances [40] and is activated by phosphorylation of the T and Y residues in the TGY motif of the activation loop (T180 and Y182 of human p38MAPKα) [41]. Analysis of p38 MAPK phosphorylation at T180 and Y182 by western blotting with phosphospecific antibodies revealed a low basal level of phosphorylation under serum-free conditions in CASMC (S4A Fig) and UASMC (S4B Fig). Serum stimulation induced a relatively slow rate of increase in phosphorylation in both cell types, which peaked at 5 min and declined towards basal levels over the 2-h incubation period.

### Serum-induced phosphorylation of HSP27

The small heat shock protein HSP27 acts as an ATP-independent chaperone in protein folding, has been implicated in cell migration and cytoskeletal architecture, and is regulated by phosphorylation at multiple sites [42], including S82, which induces polymer assembly and actin binding [43]. Analysis of HSP27 phosphorylation at S82 by western blotting with phosphospecific antibodies revealed a low basal level of phosphorylation under serum-free conditions in CASMC (S4C Fig) and UASMC (S4D Fig). Serum stimulation induced a slow increase in phosphorylation in both cell types, which peaked at 30 min and remained elevated over the 2-h incubation period.

### Knockdown of MLCK, ROCK and ZIPK

**Fig 5:**
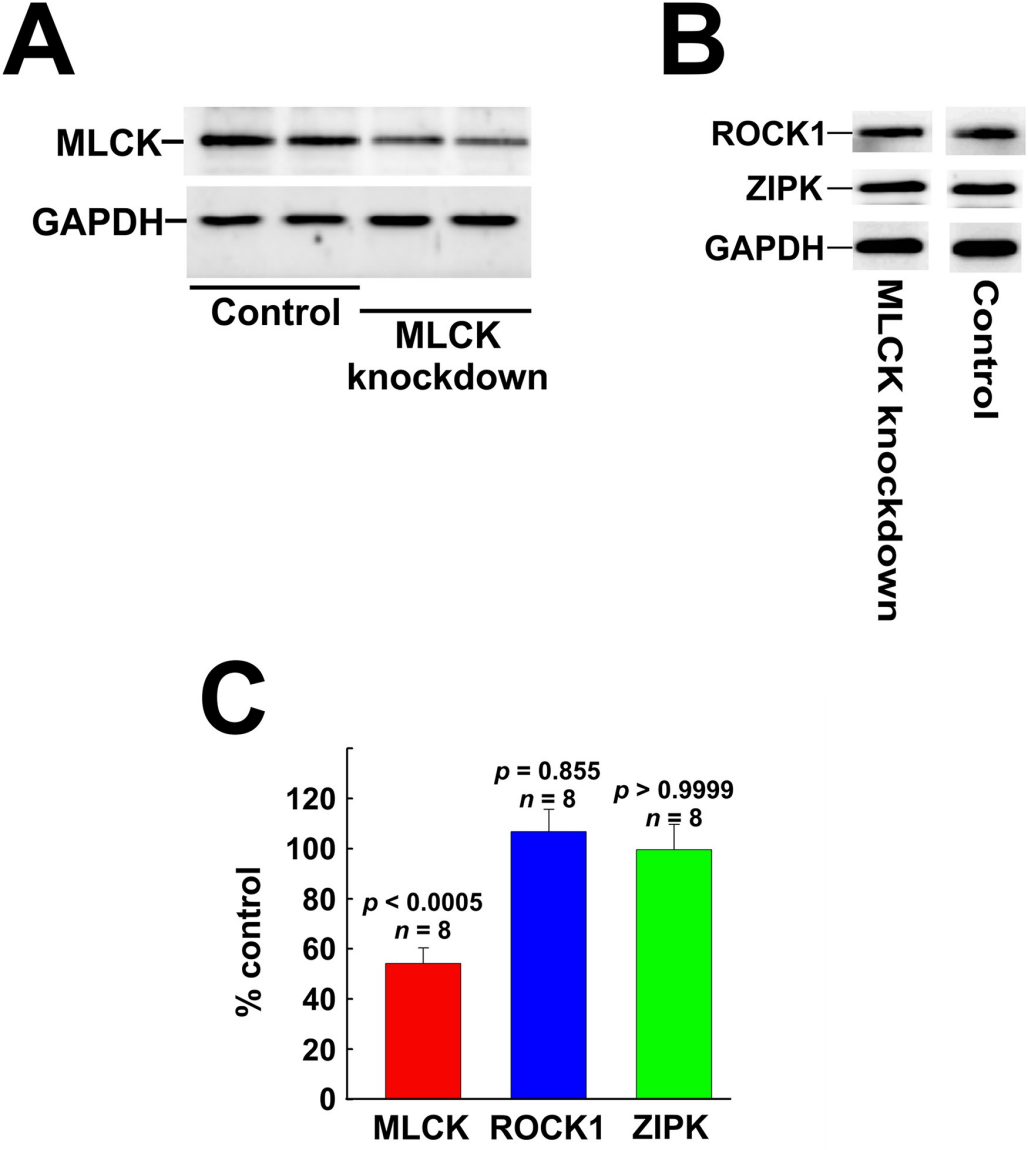
Knockdown of MLCK in cultured coronary arterial smooth muscle cells. MLCK protein was down-regulated in human CASMC by siRNA treatment. Controls were treated identically, but with negative control siRNA. (A) Representative western blots of two different knockdown experiments demonstrating siRNA-mediated down-regulation of MLCK. (B) Representative western blots showing the lack of effect of MLCK knockdown on ROCK1 or ZIPK expression in CASMC. (C) Quantitative analysis of the effects of MLCK knockdown on MLCK, ROCK1 and ZIPK expression. Statistical analysis was carried out with Dunnett’s *post hoc* test.

Transfection with siRNA was used to investigate the involvement of MLCK, ROCK and ZIPK in serum-induced phosphorylation events. Transfection of CASMC with siRNA targeting MLCK reduced MLCK protein to ~55% of control levels (cells transfected with negative control siRNA) but had no effect on ROCK1 or ZIPK levels (Fig 5).

**Fig 6:**
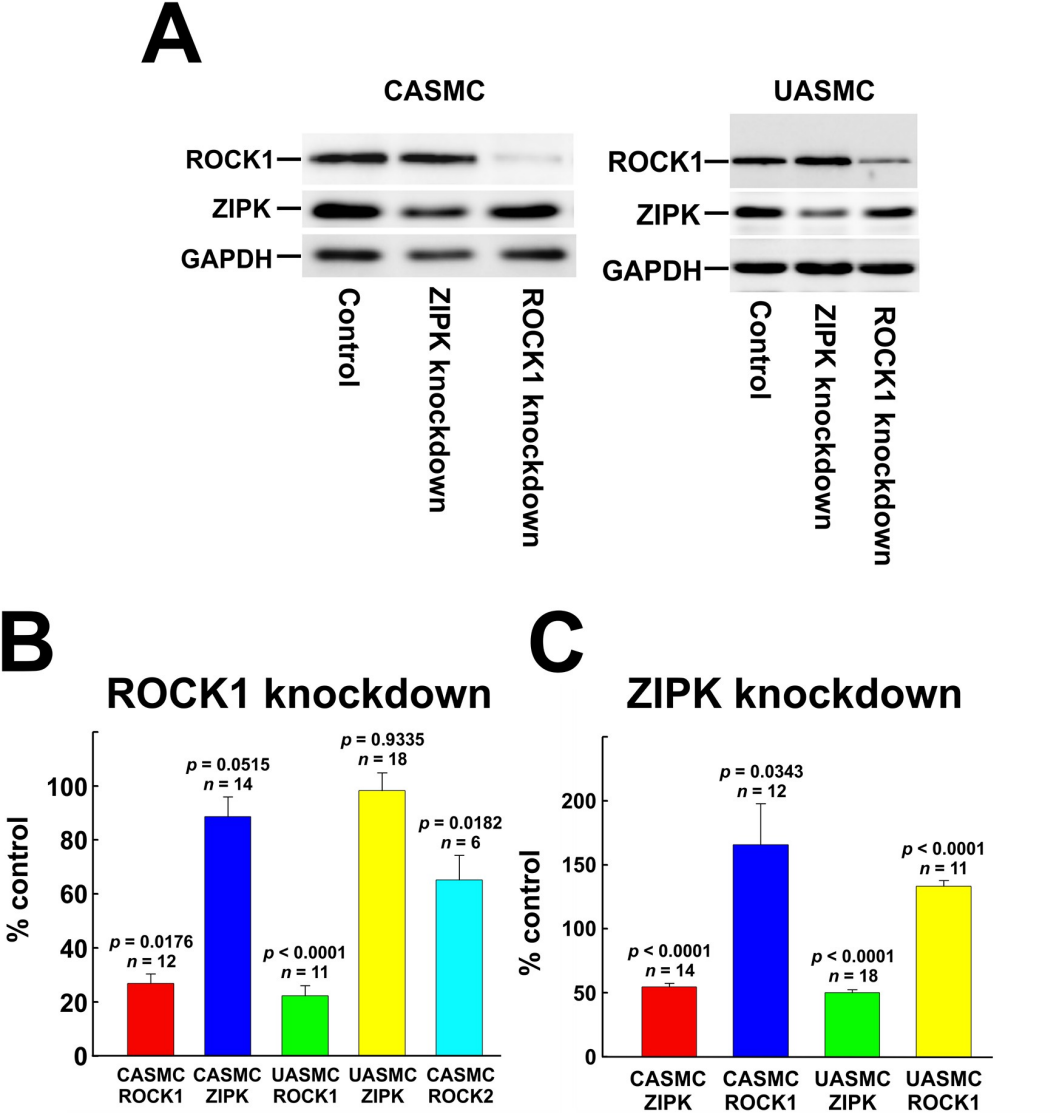
Knockdown of ROCK and ZIPK in cultured vascular smooth muscle cells. (A) Representative western blots showing the effects of ROCK1 or ZIPK knockdown on ROCK1 and ZIPK expression in CASMC and UASMC. (B) Quantification of ROCK1, ROCK2 and ZIPK levels in ROCK1 siRNA-treated cells compared to controls. (C) Quantification of ROCK1 and ZIPK levels in ZIPK siRNA-treated cells compared to controls. Statistical analysis was carried out with Dunnett’s *post hoc* test.

Transfection of CASMC and UASMC with siRNA targeting ROCK1 reduced ROCK1 protein levels to ~27 and ~22% of control (cells transfected with negative control siRNA), respectively (Fig 6), confirming our previous observations [33]. ROCK1-siRNA treatment of CASMC reduced ROCK2 protein levels to ~65% of control (Fig 6B), as we previously reported [33]. On the other hand, ROCK1 knockdown did not significantly affect ZIPK protein (Fig 6): ZIPK levels were ~89 and ~98% of controls in CASMC and UASMC, respectively, consistent with our previous observations [33].

Transfection of CASMC and UASMC with siRNA targeting ZIPK reduced ZIPK protein to ~55% and ~50% of control levels (cells transfected with negative control siRNA), respectively (Fig 6), confirming our previous observations [33]. Consistent with our previous findings [33], ZIPK knockdown was accompanied by an increase in ROCK1 protein to ~165% of control levels in CASMC and to ~133% of control levels in UASMC (Fig 6).

### Effect of MLCK knockdown and inhibition on phosphorylation of LC_20_

**Fig 7:**
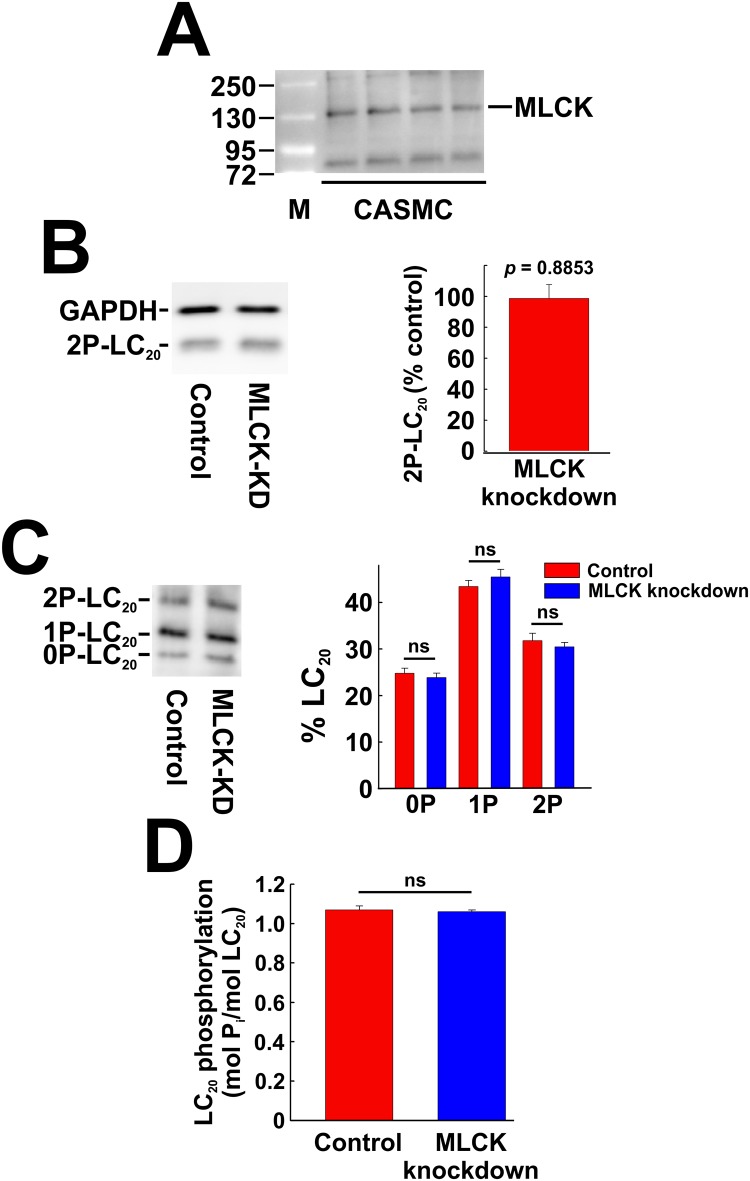
MLCK expression and the effect of knockdown of MLCK on phosphorylation of LC_20_ in coronary arterial smooth muscle cells. (A) Extracts of CASMC cultures were subjected to SDS-PAGE and western blotting with anti-MLCK. Lane “M” indicates molecular weight markers. Numbers indicate molecular weights in kDa. (B) Representative western blots with anti-2P-LC_20_ of extracts of cells treated with negative control siRNA (Control) or siRNA targeting MLCK (MLCK-KD) and then incubated with 5% FBS for 2 min (left-hand panel). GAPDH was used to normalize loading levels. Quantitative data with statistical analysis are provided in the histogram in the right-hand panel (*n* = 6; Student’s unpaired *t*-test). (C) Phos-tag SDS-PAGE of extracts of cells treated with control or MLCK-targeted siRNA following 2 min of serum stimulation (left-hand panel). The histogram (right-hand panel) shows quantitative data for unphosphorylated (0P), mono- (1P) and diphosphorylated LC_20_ (2P). “ns” indicates no significant difference between control and MLCK knockdown cells (*n* = 12; *p* > 0.05; Student’s unpaired *t*-test). (D) Quantification of LC_20_ phosphorylation stoichiometry confirmed that there was no significant effect of MLCK knockdown on serum-induced LC_20_ phosphorylation (*n* = 12; *p* = 0.9918; Student’s unpaired *t*-test).

To investigate the role of MLCK in LC_20_ phosphorylation following serum stimulation, we first examined the levels of smooth muscle MLCK in CASMC and UASMC. As shown in Fig 7A, MLCK of the expected molecular weight (130 kDa) for smooth muscle MLCK was clearly detected in CASMC by western blotting, but we could not detect the larger non-muscle isoform of 210 kDa [4]. We were unable to detect a consistent, clear signal for MLCK in UASMC, however, and therefore further experiments were carried out with CASMC. MLCK knockdown had no significant effect on LC_20_ diphosphorylation at T18 and S19 in response to serum treatment for 2 min, as shown by SDS-PAGE and western blotting with anti-2P-LC_20_ (Fig 7B). Phos-tag SDS-PAGE and western blotting with anti-LC_20_ confirmed the lack of effect of MLCK knockdown on serum-induced LC_20_ phosphorylation: phosphorylation at one and two sites and overall phosphorylation stoichiometry were unaffected by MLCK knockdown (Fig 7C and 7D).

**Fig 8:**
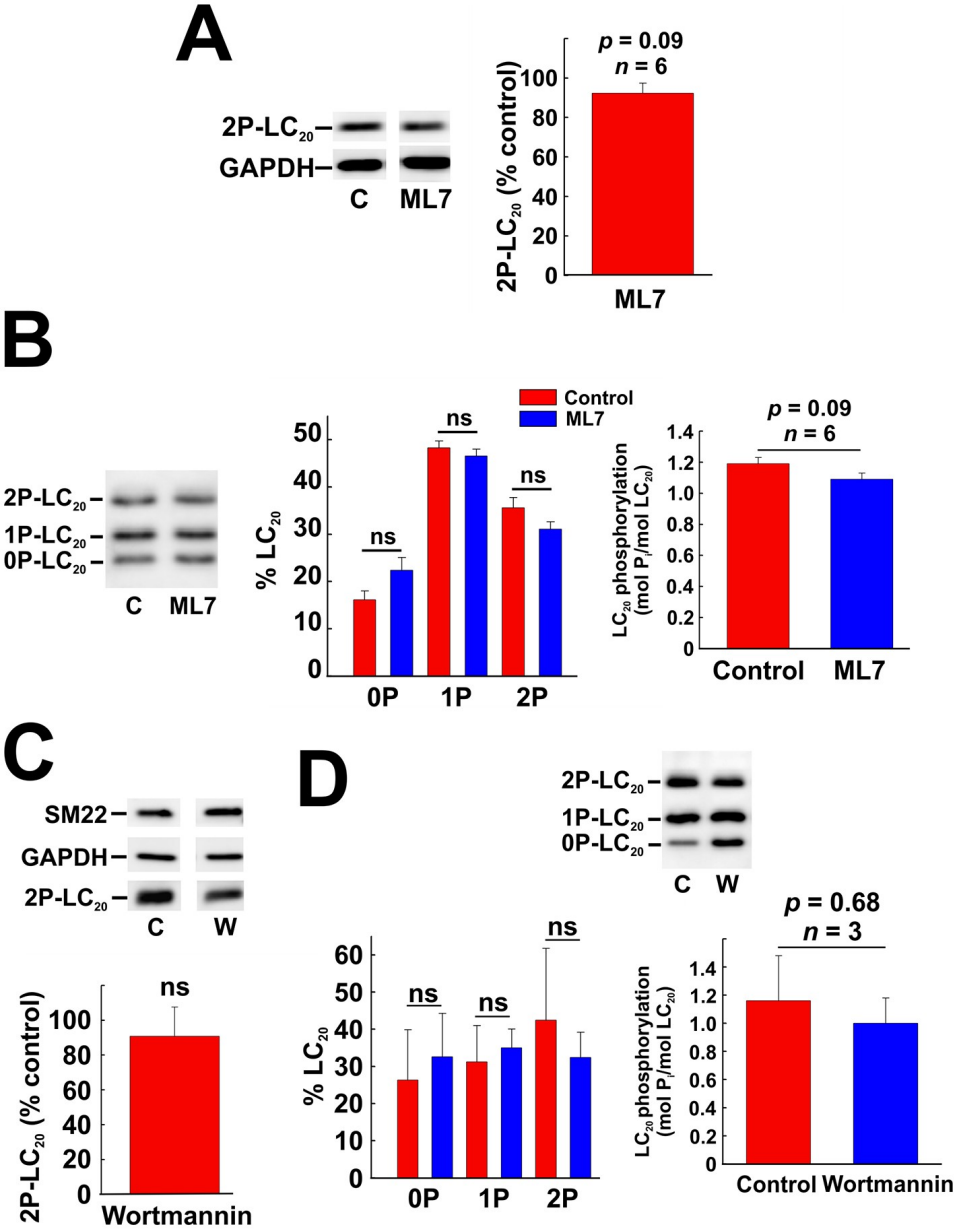
Effect of inhibition of MLCK on phosphorylation of LC_20_ in coronary arterial smooth muscle cells. (A) Effect of ML7 (10 μM) pre-treatment on serum-induced LC_20_ diphosphorylation (analysed by western blotting with anti-2P-LC_20_; left-hand panel) with quantitative analysis (right-hand panel). Statistical analysis was carried out with Student’s unpaired *t*-test. (B) Effect of ML7 (10 μM) pre-treatment on LC_20_ phosphorylation (analysed by Phos-tag SDS-PAGE and western blotting with anti-LC_20_) following 2 min of serum stimulation. Controls (C) were incubated with vehicle rather than ML7. A representative western blot is shown in the left-hand panel, quantification of unphosphorylated, mono- and diphosphorylated LC_20_ in the middle panel and overall phosphorylation stoichiometry in the right-hand panel (*n* = 6). Statistical analysis was carried out with Student’s unpaired *t*-test. (C) Effect of wortmannin (1 μM) pre-treatment on serum-induced LC_20_ diphosphorylation (analysed by western blotting with anti-2P-LC_20_; upper panel) with quantitative analysis (*n* = 3; lower panel). (D) Effect of wortmannin (1 μM) pre-treatment on serum-induced LC_20_ diphosphorylation (analysed by Phos-tag SDS-PAGE with anti-LC_20_; upper panel) with quantitative analysis (lower panels). “ns” denotes *p* > 0.05 (Student’s unpaired *t*-test).

The MLCK inhibitors, ML7 and wortmannin, also had no significant effect on LC_20_ phosphorylation at T18 and S19 in response to serum treatment. SDS-PAGE and western blotting with anti-2P-LC_20_ indicated that serum-induced LC_20_ diphosphorylation was unaffected by ML7 (Fig 8A). Phos-tag SDS-PAGE analysis confirmed that there was no significant difference between serum-induced mono- or diphosphorylation of LC_20_ in the absence or presence of ML7 (Fig 8B). Likewise, SDS-PAGE and western blotting with anti-2P-LC_20_ indicated that serum-induced LC_20_ diphosphorylation was unaffected by wortmannin (Fig 8C), which was confirmed by Phos-tag SDS-PAGE analysis (Fig 8D). In control experiments, we verified the membrane permeability and efficacy of ML7 by demonstrating inhibition of U46619 (thromboxane A_2_ mimetic)-induced contraction of de-endothelialized rat caudal arterial smooth muscle, as described previously [44]. We also verified that wortmannin (an inhibitor of PI 3-kinase as well as MLCK) is membrane permeant and effective since it inhibited phosphorylation of Akt, a PI 3-kinase substrate (S5 Fig).

**Fig 9:**
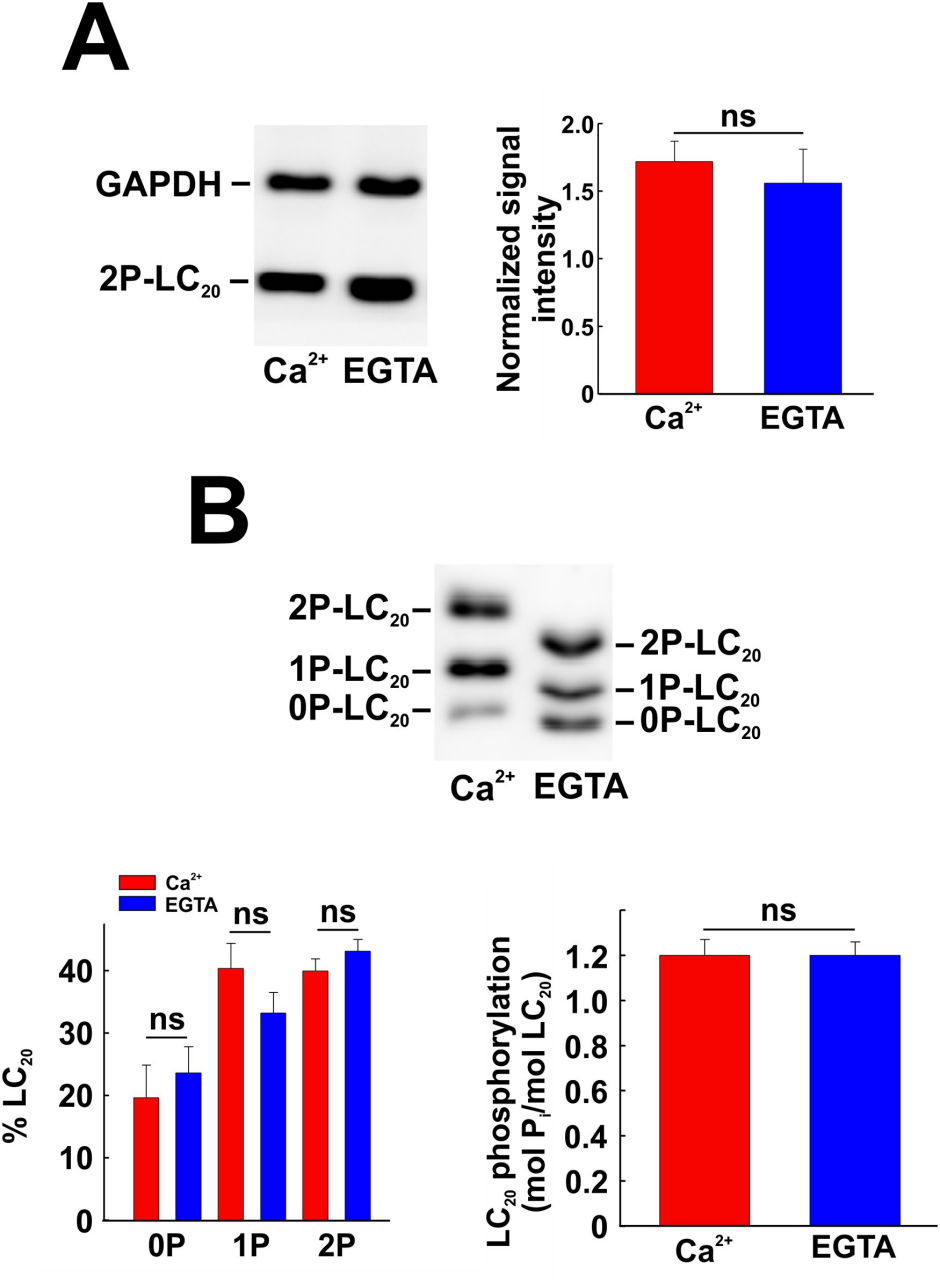
Effect of EGTA treatment on serum-induced LC_20_ phosphorylation. CASMC were incubated in medium containing Ca^2+^ (2.5 mM) or EGTA (5 mM) and treated with serum for 2 min prior to SDS-PAGE and western blotting with anti-2P-LC_20_ (A; left-hand panel) or Phos-tag SDS-PAGE and western blotting with anti-LC_20_ (B; upper panel). Quantitative data are depicted in the corresponding histograms in panels A and B (*n* = 3 in panel A and *n* = 6 in panel B). “ns” denotes *p* > 0.05 (Student’s unpaired *t*-test).

Finally, chelation of Ca^2+^ with EGTA to remove extracellular Ca^2+^ and deplete intracellular stores had no significant effect on serum-induced LC_20_ phosphorylation at T18 and S19. From western blotting with anti-diP-LC_20_, there was no significant difference between the normalized signal intensities for diP-LC_20_ from cells treated with serum in the presence of Ca^2+^ or EGTA (Fig 9A). From Phos-tag SDS-PAGE and western blotting with anti-LC_20_, LC_20_ phosphorylation stoichiometry was identical in the presence of Ca^2+^ and EGTA (Fig 9B). EGTA treatment affected the mobility of LC_20_ species during Phos-tag SDS-PAGE (Fig 9B) but did not affect the phosphorylation stoichiometry. We are confident that the high concentration of EGTA used (5 mM) does effectively deplete the endogenous calcium stores in smooth muscle cells since we have shown that a lower EGTA concentration (2 mM) abolished both caffeine-induced and U-46619-induced contraction of de-endothelialized rat caudal arterial smooth muscle and U-46619-induced LC_20_ phosphorylation, which occurred exclusively at S19 [44].

### Effect of ROCK knockdown on phosphorylation of LC_20_, MYPT1 and Par-4

**Fig 10:**
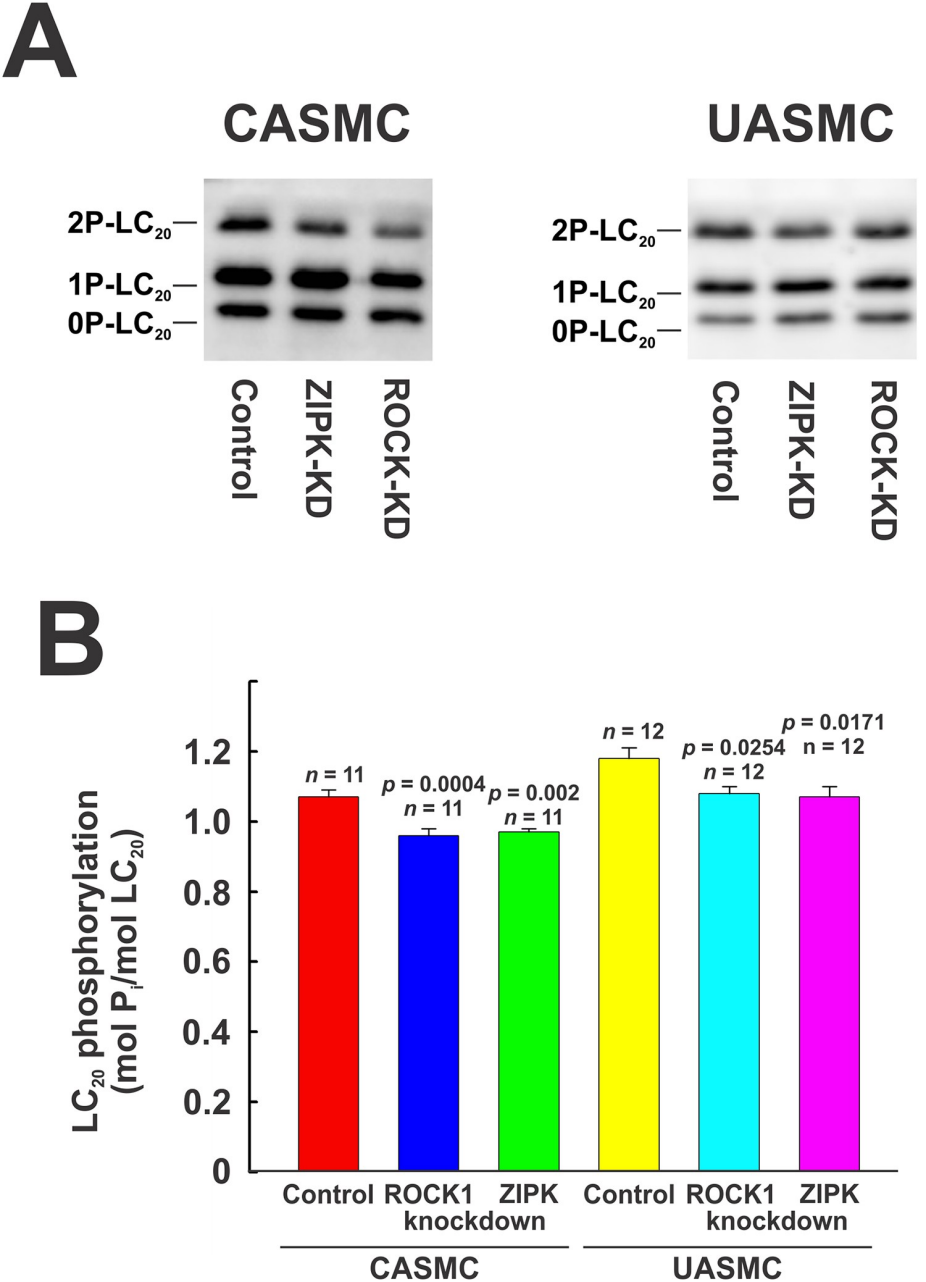
Effect of knockdown of ROCK1 or ZIPK on phosphorylation of LC_20_ in human arterial smooth muscle cells assessed by Phos-tag SDS-PAGE. ROCK1 or ZIPK was down-regulated in CASMC and UASMC by siRNA treatment. Controls were treated identically, but with negative control siRNA. Cells were serum starved overnight and treated with 5% FBS for 2 min prior to lysis in Laemmli sample buffer for Phos-tag SDS-PAGE and western blotting with anti-LC_20_. Representative western blots are shown in panel A (CASMC and UASMC) and cumulative quantitative data are provided in the histograms in panel B. Data in panel B are presented as LC_20_ phosphorylation stoichiometry and values represent the mean ± SEM with the indicated *p* and *n* values with *p* < 0.05 considered statistically significant (Dunnett’s *post ho*c test).

**Fig 11:**
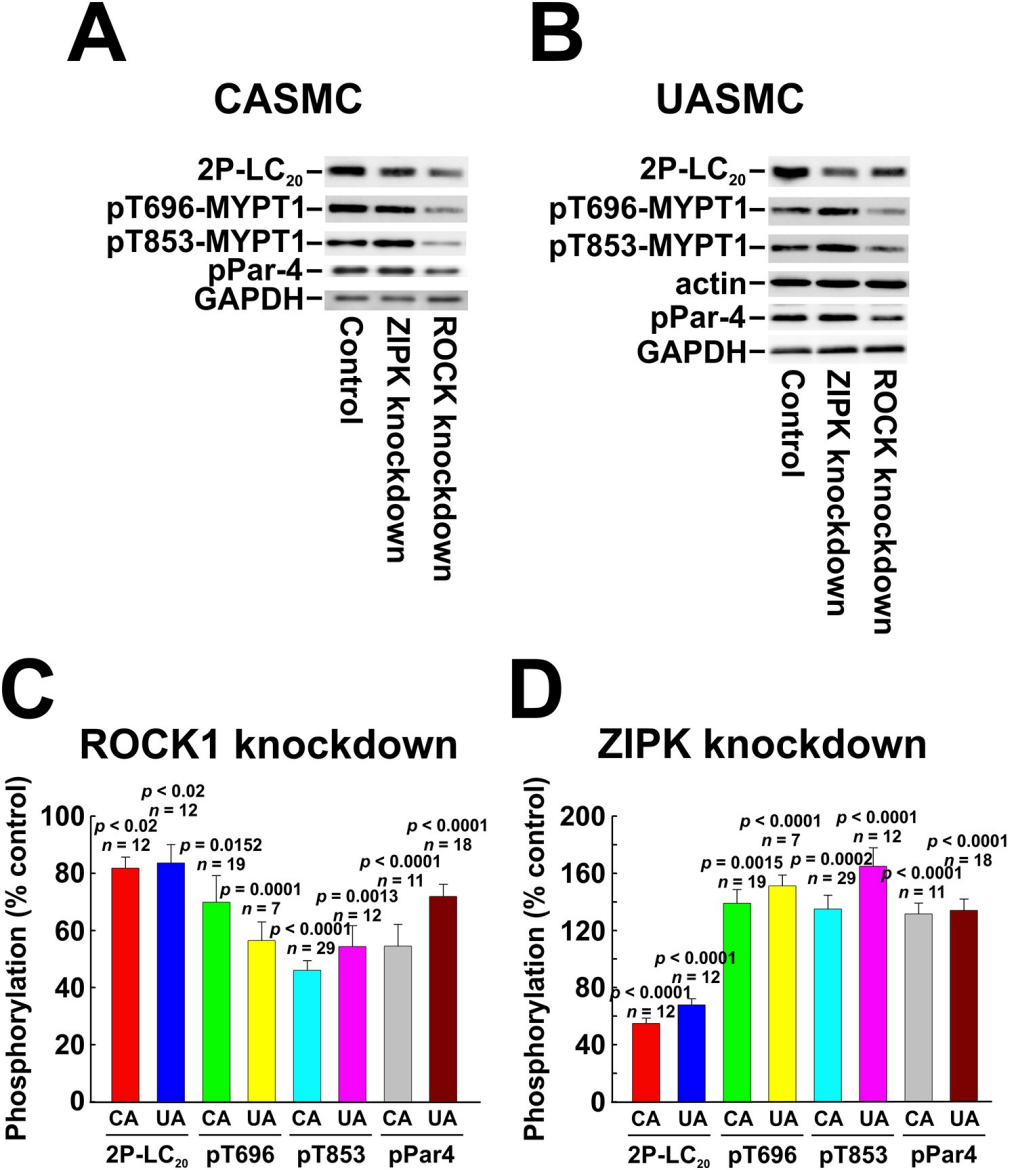
Effect of knockdown of ROCK1 or ZIPK on phosphorylation of LC_20_, MYPT1 and Par-4 in human arterial smooth muscle cells. ROCK1 or ZIPK was down-regulated in CASMC and UASMC by siRNA treatment. Controls were treated identically, but with negative control siRNA. Cells were serum starved overnight and treated with 5% FBS for 2 min prior to lysis in Laemmli sample buffer for SDS-PAGE and western blotting. GAPDH and actin were used as loading controls; each control shown is for the blot(s) above it. Representative western blots are shown in panels A (CASMC) and B (UASMC) and cumulative quantitative data are provided in the histograms (C, D). Data in panels C and D are presented as % control and values represent the mean ± SEM with the indicated *p* and *n* values with *p* < 0.05 considered statistically significant (Dunnett’s *post ho*c test).

ROCK-knockdown and control cells were serum starved overnight, treated with 5% FBS for 2 min and lysed for SDS-PAGE and western blotting. Down-regulation of ROCK correlated with significant reductions in serum-induced diphosphorylation of LC_20_ (Fig 10), of MYPT1 at T696 and T853, and of Par-4 at T163 in both CASMC and UASMC (Fig 11).

### Effect of ROCK inhibition on phosphorylation of LC_20_, MYPT1 and Par-4

**Fig 12:**
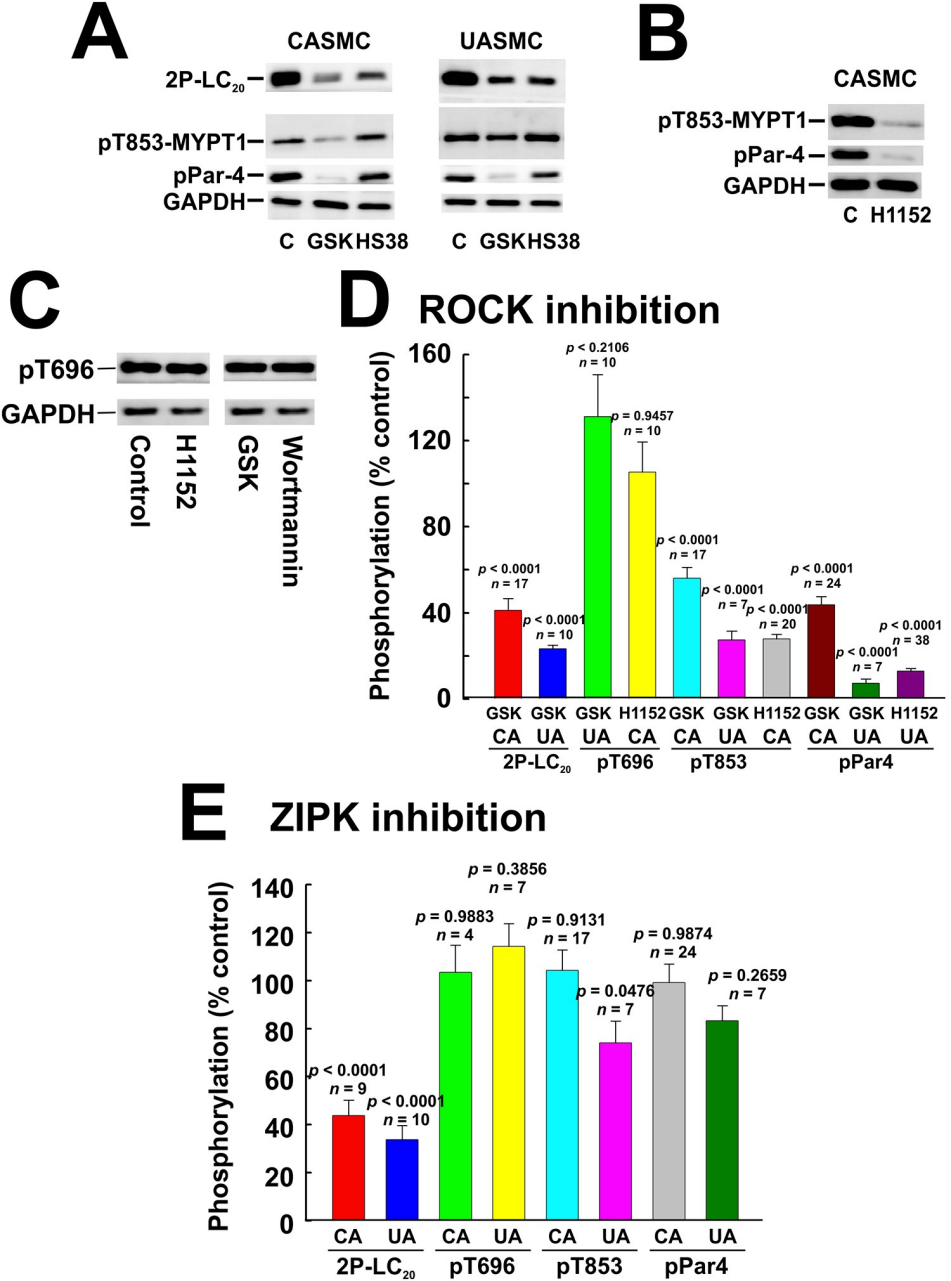
Effect of inhibition of ROCK or ZIPK on phosphorylation of LC_20_, MYPT1 and Par-4 in human arterial smooth muscle cells. CASMC or UASMC were serum starved overnight, incubated for 40 min with GSK429286A (GSK; 1 μM), HS38 (10 μM), H1152 (1 μM) or vehicle control (C) and treated with 5% FBS for 2 min prior to lysis in Laemmli sample buffer for SDS-PAGE and western blotting with anti-2P-LC_20_, anti-pT853-MYPT1 or anti-pT163-Par-4 (A), with anti-pT853-MYPT1 or anti-pT163-Par-4 (B) or with anti-pT696-MYPT1 (C). Phosphorylated bands were quantified by scanning densitometry and normalized to the loading control (GAPDH). (D, E) Quantitative data showing the effects of ROCK inhibition with GSK429286A or H1152 (D) or ZIPK inhibition with HS38 (E) on serum-induced phosphorylation of the indicated proteins. Data are presented as % control and values represent the mean ± SEM with the indicated *p* and *n* values with *p* < 0.05 considered statistically significant (Dunnett’s *post ho*c test).

**Fig 13:**
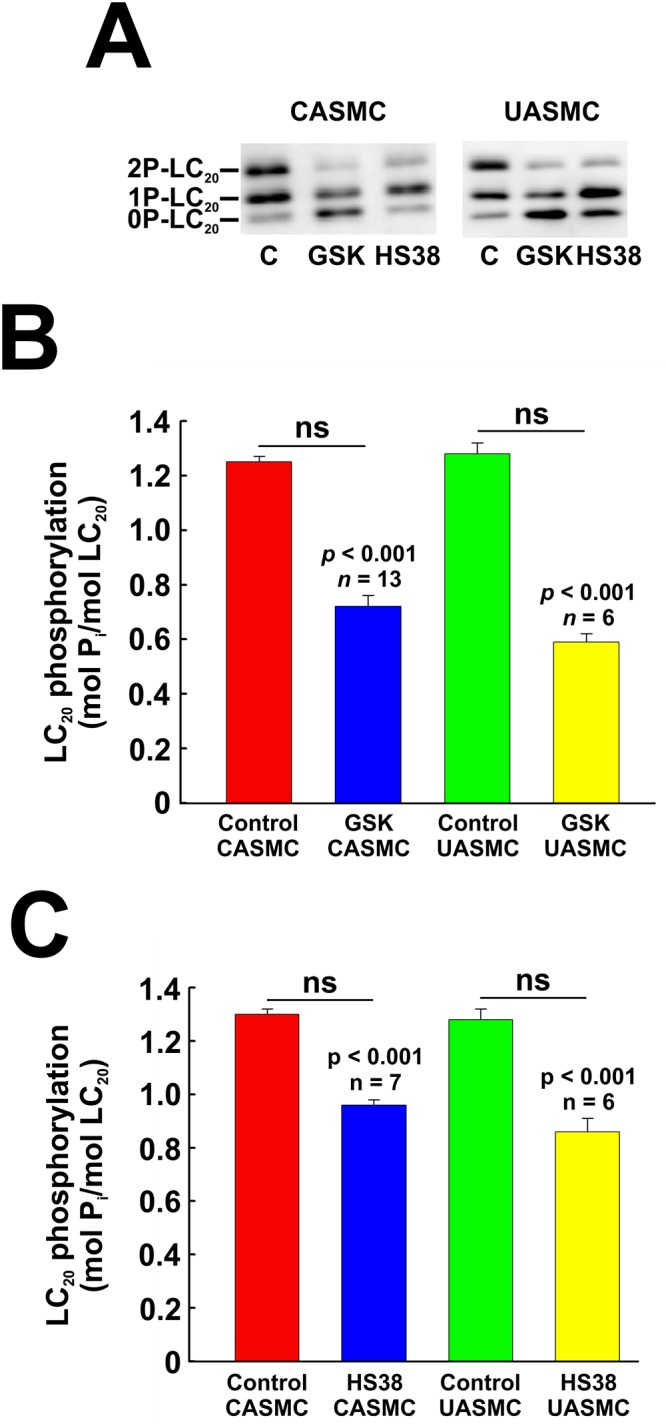
Effect of inhibition of ROCK or ZIPK on phosphorylation of LC_20_ assessed by Phos-tag SDS-PAGE. CASMC or UASMC were serum starved overnight, incubated for 40 min with GSK429286A (GSK; 1 μM), HS38 (10 μM) or vehicle control (C) and treated with 5% FBS for 2 min prior to lysis in Laemmli sample buffer for Phos-tag SDS-PAGE and western blotting with anti-LC_20_ (A). (B, C) Unphosphorylated (0P-LC_20_), monophosphorylated (1P-LC_20_) and diphosphorylated LC_20_ (2P-LC_20_) bands were quantified by scanning densitometry. Data are presented as LC_20_ phosphorylation stoichiometry and values represent the mean ± SEM with the indicated *p* and *n* values with *p* < 0.05 considered statistically significant (Dunnett’s *post ho*c test).

Pre-treatment of cells with the ROCK inhibitor GSK429286A (1 μM) had a pronounced inhibitory effect on serum-induced LC_20_ diphosphorylation in both CASMC and UASMC (Fig 12A and 12D). Phos-tag SDS-PAGE confirmed that LC_20_ phosphorylation stoichiometry was reduced by ROCK inhibition in CASMC and UASMC (Fig 13A and 13B). ROCK inhibition by GSK429286A also markedly reduced the serum-induced increase in phosphorylation of MYPT1 at T853 and of Par-4 at T163, but had no significant inhibitory effect on MYPT1 phosphorylation at T696 (Fig 12A and 12D). Similarly, ROCK inhibition by H1152 (0.2 μM) in CASMC had no effect on MYPT1 phosphorylation at T696 but markedly reduced MYPT1 phosphorylation at T853 and Par-4 phosphorylation at T163 (Fig 12B, 12C and 12D).

### Effect of ZIPK knockdown on phosphorylation of LC_20_, MYPT1 and Par-4

ZIPK-knockdown and control cells were serum starved overnight, treated with 5% FBS for 2 min and lysed for SDS-PAGE and western blotting. LC_20_ diphosphorylation was reduced to ~60% of control in CASMC and UASMC (Fig 11A, 11B and 11D). Phos-tag SDS-PAGE confirmed that ZIPK knockdown caused a small, but significant reduction in serum-induced LC_20_ phosphorylation in both CASMC and UASMC (Fig 10). ZIPK knockdown in both CASMC and UASMC increased MYPT1 phosphorylation at T696 and T853 and Par-4 phosphorylation at T163 (Fig 11A, 11B and 11D). The increases in MYPT1 and Par-4 phosphorylation could be accounted for by the increase in ROCK protein levels induced by ZIPK knockdown shown earlier (Fig 6).

### Effect of ZIPK inhibition on phosphorylation of LC_20_, MYPT1 and Par-4

ZIPK inhibition by HS38 [28] attenuated the increase in LC_20_ diphosphorylation induced by treatment of CASMC and UASMC with 5% FBS for 2 min, but had no effect on the phosphorylation of MYPT1 at T696 or T853 or of Par-4 at T163 (Fig 12A and 12E). Phos-tag SDS-PAGE confirmed that HS38 reduced the serum-induced phosphorylation stoichiometry of LC_20_ in CASMC and UASMC (Fig 13A and 13C).

### Serum-induced phosphorylation of ZIPK and the effect of ROCK and ZIPK knockdown and inhibition

**Fig 14:**
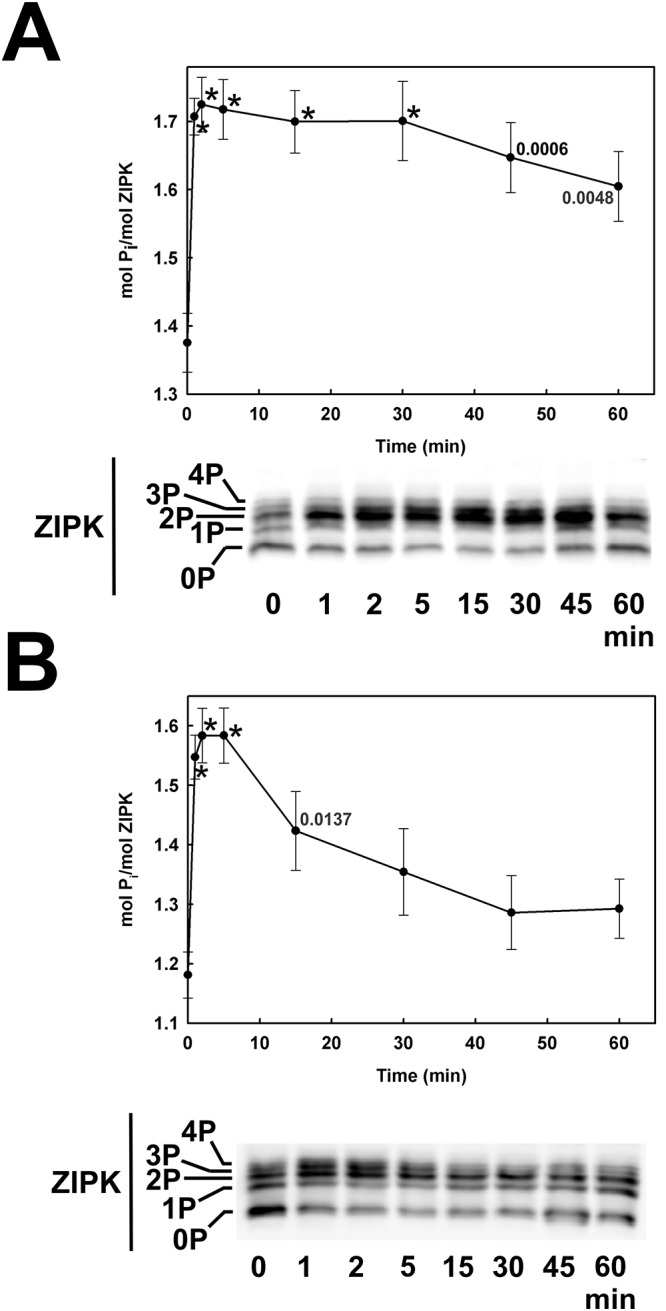
Time courses of serum-induced ZIPK phosphorylation in human arterial smooth muscle cells. Human coronary (A) and umbilical arterial smooth muscle cells (B) were treated with serum at time zero, as described in the legend to Fig 1. Cells were lysed in Laemmli sample buffer at the indicated times and subjected to Phos-tag SDS-PAGE and western blotting with anti-ZIPK as described in the Materials and Methods section. Representative western blots are shown below the time courses of ZIPK phosphorylation stoichiometry. Data are presented as mean ± SEM (*n* = 9 in panel A; *n* = 11 in panel B). Statistically significant differences from ZIPK phosphorylation stoichiometry at time zero are denoted by * (*p* < 0.0001) or by the actual *p* values (Dunnett’s *post hoc* test).

**Fig 15:**
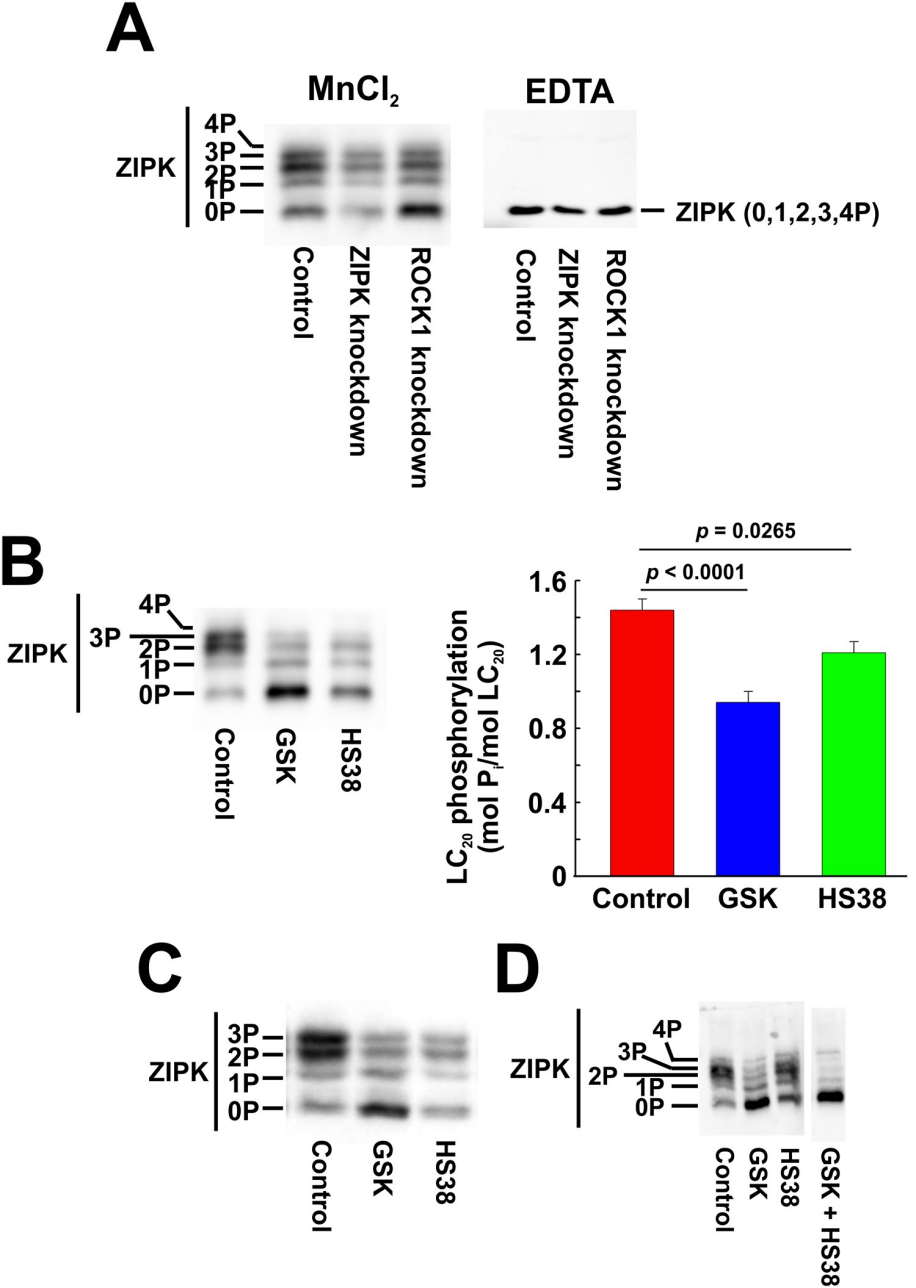
Effect of ROCK1 and ZIPK knockdown and inhibition on serum-induced ZIPK phosphorylation. (A) ROCK1 or ZIPK expression was down-regulated in CASMC by siRNA treatment. Controls were treated identically, but with negative control siRNA. Cells were serum starved overnight and treated with 5% FBS for 2 min prior to lysis in Laemmli sample buffer for Phos-tag SDS-PAGE and western blotting with anti-ZIPK. Electrophoresis was carried out in the presence of MnCl_2_ or EDTA as indicated. CASMC (B) or UASMC (C) were serum starved overnight and treated with 5% FBS for 2 min in the absence (control) or presence of GSK429286A (GSK; 1 μM) or HS38 (10 μM) prior to lysis in Laemmli sample buffer for Phos-tag SDS-PAGE and western blotting with anti-ZIPK. Representative blots are shown (*n* = 11 in panel A; *n* = 7 in panel B; *n* = 4 in panel C; *n* = 3 in panel D). Statistically significant differences in LC_20_ phosphorylation stoichiometry from control are denoted by the actual *p* values (Dunnett’s *post hoc* test; panel B).

ZIPK phosphorylation, reflecting activation of the kinase [45], was assessed by Phos-tag SDS-PAGE to separate phosphorylated and unphosphorylated forms of the enzyme, which were then detected by western blotting with a pan-ZIPK antibody. Under serum-free conditions, ZIPK was partially phosphorylated in CASMC and UASMC with low levels of phosphorylation at one, two, three and four sites (time zero samples in Fig 14A and 14B, respectively). Serum treatment induced a rapid increase in ZIPK phosphorylation, particularly of the diphosphorylated protein, which was maintained for ≥ 1 h in CASMC (Fig 14A) but transient in UASMC (Fig 14B). Knockdown of ROCK1 had a significant inhibitory effect on serum-induced ZIPK phosphorylation, while ZIPK siRNA treatment reduced ZIPK levels but did not affect the protein’s phosphorylation in response to serum treatment (Fig 15A, left-hand panel). Chelation of divalent cations with EDTA resulted in a single band of ZIPK corresponding to the migration of the unphosphorylated protein (Fig 15A, right-hand panel) confirming that the slower migrating bands observed in the presence of MnCl_2_ (Fig 15A, left-hand panel) are indeed phosphorylated forms of ZIPK. Inhibitors of ROCK (GSK429286A) and ZIPK (HS38) inhibited the serum-induced phosphorylation of ZIPK in CASMC (Fig 15B) and UASMC (Fig 15C) suggesting that ROCK phosphorylates ZIPK, and ZIPK can autophosphorylate. The combination of ROCK and ZIPK inhibitors prevented ZIPK phosphorylation induced by serum stimulation and almost abolished the basal level of ZIPK phosphorylation in the absence of serum stimulation of UASMC (Fig 15D).

### Effects of kinase knockdown and inhibition on LC_20_ phosphorylation under serum-free conditions

**Fig 16:**
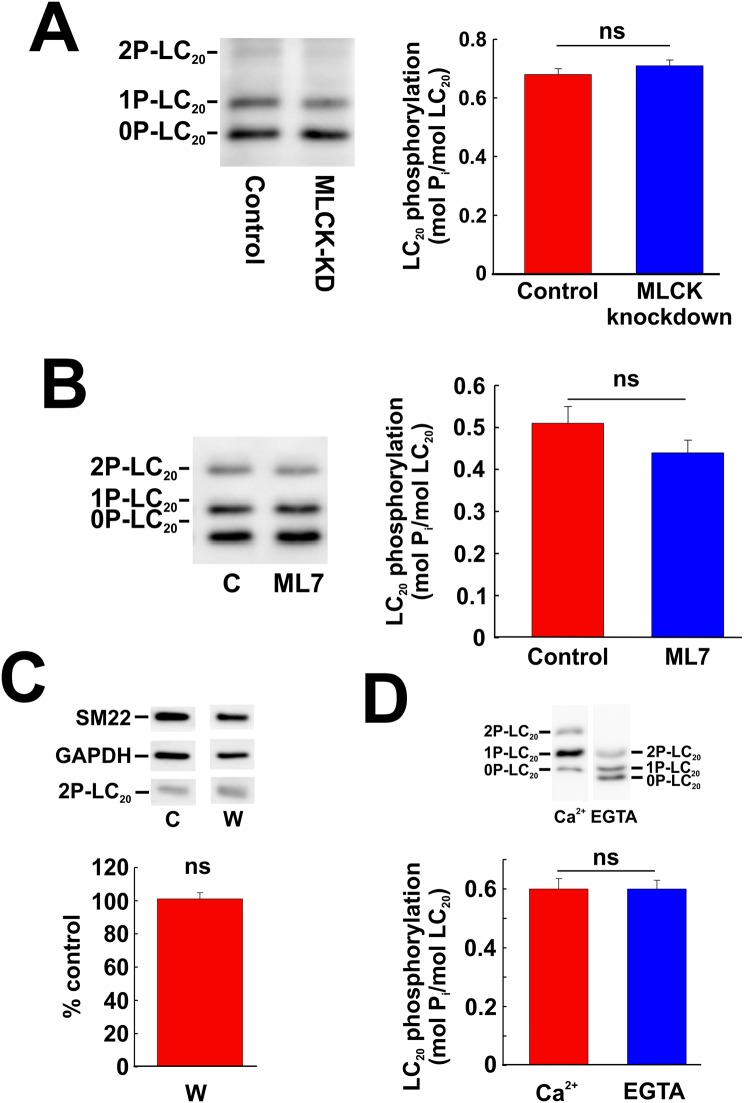
Effect of MLCK knockdown or inhibition on LC_20_ phosphorylation under serum-free conditions. (A) MLCK was down-regulated in CASMC by siRNA treatment. Controls were treated identically, but with negative control siRNA. Cells were serum starved overnight prior to lysis in Laemmli sample buffer for Phos-tag SDS-PAGE and western blotting with anti-LC_20_. A representative western blot (left-hand panel) and cumulative quantitative data (right-hand panel) are shown. Data are presented as LC_20_ phosphorylation stoichiometry and values represent the mean ± SEM. “ns” denotes *p* > 0.05 (*n* = 10; Student’s unpaired *t*-test). (B) Effect of ML7 (10 μM) treatment on LC_20_ phosphorylation (analysed by Phos-tag SDS-PAGE and western blotting with anti-LC_20_) in the absence of serum stimulation. Controls (C) were incubated with vehicle rather than ML7. A representative western blot is shown in the left-hand panel and overall phosphorylation stoichiometry in the right-hand panel. “ns” denotes *p* > 0.05 (*n* = 6; Dunnett’s *post hoc* test). (C) Effect of wortmannin (1 μM) treatment on LC_20_ diphosphorylation (analysed by western blotting with anti-2P-LC_20_: representative western blots are shown in the upper panel with cumulative quantitative data in the lower panel. “ns” denotes *p* > 0.05 (*p* = 0.8633; *n* = 11; Student’s unpaired *t*-test). (D) Effect of chelation of Ca^2+^ with EGTA on LC_20_ phosphorylation under serum-free conditions. CASMC were incubated in medium containing Ca^2+^ (2.5 mM) or EGTA (5 mM) under serum-free conditions prior to Phos-tag SDS-PAGE and western blotting with anti-LC_20_. A representative western blot is shown in the upper panel with quantitative data depicted in the lower panel. “ns” denotes *p* > 0.05 (Student’s unpaired *t*-test).

**Fig 17:**
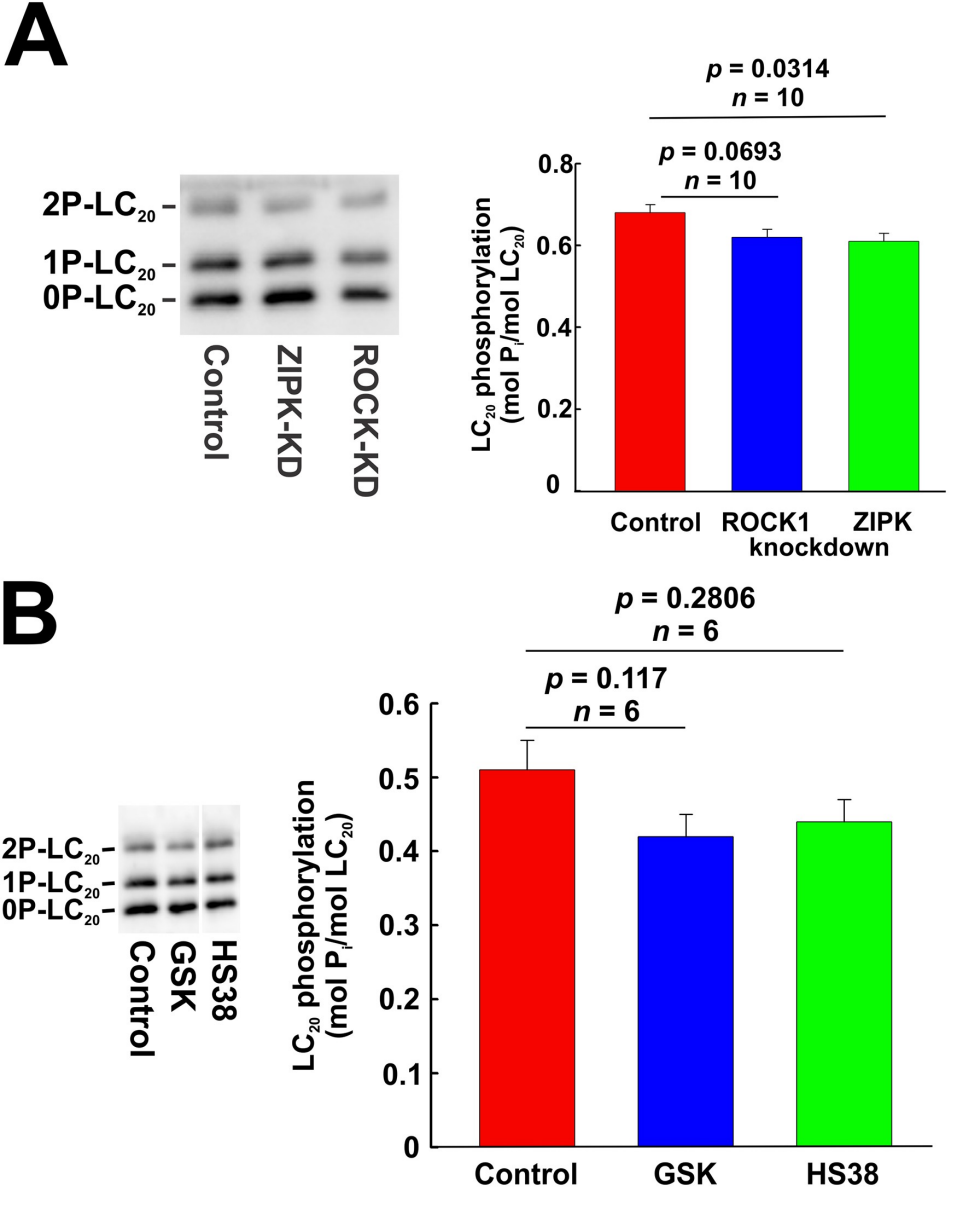
Effect of knockdown and inhibition of ROCK1 and ZIPK on LC_20_ phosphorylation under serum-free conditions assessed by Phos-tag SDS-PAGE. (A) ROCK1 or ZIPK was down-regulated by siRNA treatment. Controls were treated identically, but with negative control siRNA. Cells were serum starved overnight prior to lysis in Laemmli sample buffer for Phos-tag SDS-PAGE and western blotting with anti-LC_20_. A representative western blot is shown in the left-hand panel with cumulative quantitative data on LC_20_ phosphorylation stoichiometry in the right-hand panel. Statistical analysis was carried out with Dunnett’s *post hoc* test. (B) The effect of inhibition of ROCK or ZIPK on LC_20_ phosphorylation in UASMC assessed by Phos-tag SDS-PAGE and western blotting with anti-LC_20_. A representative western blot is shown in the left-hand panel with cumulative quantitative data on LC_20_ phosphorylation stoichiometry in the right-hand panel. Statistical analysis was carried out with Dunnett’s *post hoc* test.

As shown in Fig 1, there is a basal level of LC_20_ diphosphorylation in serum-starved cultured human vascular smooth muscle cells. We used a combination of siRNA-mediated knockdown and pharmacological inhibition to evaluate the roles of MLCK, ROCK and ZIPK in the regulation of LC_20_ phosphorylation in the absence of serum stimulation. Phos-tag SDS-PAGE and western blotting with anti-LC_20_ showed no effect of MLCK knockdown on LC_20_ phosphorylation under serum-free conditions (Fig 16A). The MLCK inhibitors, ML7 and wortmannin, also had no significant effect on LC_20_ phosphorylation at T18 and S19 under serum-free conditions (Fig 16B and 16C, respectively). Furthermore, chelation of Ca^2+^ with EGTA had no effect on LC_20_ phosphorylation at T18 and S19 in the absence of serum stimulation (Fig 16D). Finally, ROCK1 knockdown (Fig 17A) or inhibition by GSK429286A (Fig 17B) or ZIPK knockdown (Fig 17A) or inhibition with HS38 (Fig 17B) under serum-free conditions did not have a significant effect on LC_20_ phosphorylation under serum-free conditions.

## Discussion

The contractile response of smooth muscle can be enhanced or sustained by the process of Ca^2+^ sensitization, which involves a reduction in MLCP activity leading to an increase in the MLCK: MLCP activity ratio [46,47] and sometimes involves diphosphorylation of LC_20_ at T18 and S19 [9]. Similar findings have been reported in cultured smooth muscle and non-muscle cells [11–13]. In this study, we focused on two protein kinases, ROCK [48,49] and ZIPK [50–52], that have been implicated in the process of Ca^2+^ sensitization and LC_20_ diphosphorylation [15,18,20,53–55]. Furthermore, while LC_20_ is unlikely to be a direct physiological substrate of ROCK in smooth muscle tissue [56–62], there is evidence of direct phosphorylation of LC_20_ at T18 and S19 by ROCK in cultured cells [63,64]. Likewise, ZIPK has been shown to phosphorylate LC_20_ at both T18 and S19 [18–20,55]. Both kinases are Ca^+^-independent, unlike MLCK which requires Ca^2+^/calmodulin for activity and phosphorylates LC_20_ exclusively at S19 [9].

ROCK has been shown to phosphorylate the myosin targeting subunit of MLCP (MYPT1) at T696 and T853 (human MYPT1 numbering; GenBank ID number: 289142212) resulting in decreased phosphatase activity [46,48,65–70]. T696 can be phosphorylated by several other protein kinases [54,69] and it appears to be constitutively phosphorylated, i.e. significantly phosphorylated in the absence of stimulation [71]. T853 is more commonly phosphorylated in response to stimulation [44,71]. ROCK can also phosphorylate Par-4 at T163, which abolishes the interaction of Par-4 with MYPT1 and exposes T696 and T853 of MYPT1 to endogenous kinases, resulting in inhibition of MLCP activity, increased LC_20_ phosphorylation and contraction [72]. While this regulatory mechanism was demonstrated in cultured cells, its operation in vascular smooth muscle tissue has been questioned [73].

ZIPK (DAPK3) is a member of the death-associated protein kinase (DAPK) family, which includes DAPK1, DAPK2, DAP-related protein kinase 1 (DRAK1) and DRAK2 [74,75]. Unlike other DAPK family members, ZIPK lacks a calmodulin-binding site and its activity is independent of Ca^2+^ [76]. Seven phosphorylation sites have been identified in ZIPK: T180, T225, T265, T299, T300, T306 and S311 [77]. Phosphorylation at T299, T306 and T311 has little effect on ZIPK activity, but phosphorylation at T180 (within the kinase activation loop), T225 (in the substrate-binding groove) and T265 (in kinase subdomain X) is essential for full kinase activation [45]. ROCK also phosphorylates ZIPK at multiple sites leading to its activation [45,77–79], and our results provide additional evidence for the integration of ZIPK and ROCK signaling networks at the cellular level.

Arterial smooth muscle cells undergo dedifferentiation when cultured, which involves transition from a non-proliferative, contractile phenotype to a proliferative, motile phenotype [80]. These changes reflect similar changes occurring in arterial smooth muscle cells *in situ* that contribute to cardiovascular diseases such as atherosclerosis [81]. The major goals of this study were to characterize the time courses of serum-induced phosphorylation events relevant to contractility, migration and cytokinesis of human cultured arterial smooth muscle cells [48], with an emphasis on LC_20_ diphosphorylation and the kinases responsible, directly and indirectly, for its phosphorylation. We used two experimental approaches to investigate ROCK and ZIPK as likely candidates in the diphosphorylation of LC_20_ as well as MYPT1 and Par-4 phosphorylation in response to serum treatment: (i) siRNA-mediated knockdown of ROCK and ZIPK, and (ii) inhibition of kinase activity by well-characterized and selective inhibitors (GSK429286A and H1152 for ROCK and HS38 for ZIPK).

Exposure of serum-starved cells to serum resulted in the rapid phosphorylation of LC_20_ at T18 and S19 (Figs 1 and 2). Similar time courses of serum-induced phosphorylation of MYPT1 at T696 and T853 (Fig 3) and of Par-4 at T163 (Fig 4) were observed, both of which result in inhibition of MLCP activity [72,82], suggesting that the rapid diphosphorylation of LC_20_ is a consequence of activation of a kinase(s) that directly phosphorylates LC_20_ with the concomitant inhibition of MLCP activity. Furthermore, LC_20_ phosphorylation at both T18 and S19 reduces the rate of LC_20_ dephosphorylation by MLCP compared to that of LC_20_ phosphorylated exclusively at S19 [10]. This helps to explain the observed sustained phosphorylation of LC_20_.

The most obvious candidate kinase for the serum-induced phosphorylation of LC_20_ was MLCK itself. While S19 is the site of phosphorylation responsible for smooth muscle contraction, MLCK is well known to be capable of phosphorylating T18 as well [5]. As noted in the Introduction, however, this has only been demonstrated *in vitro* with purified MLCK at supra-physiological concentrations relative to the LC_20_ substrate [5,6] and there is plenty of evidence to support the conclusion that *in situ* and under physiological conditions MLCK does not phosphorylate T18 [7,8,83]. Nevertheless, we investigated the potential role of MLCK in serum-induced LC_20_ phosphorylation in CASMC and found that: (i) western blot analysis confirmed the expression of smooth muscle MLCK in CASMC (Fig 7A), (ii) MLCK knockdown had no effect on LC_20_ phosphorylation at T18 or S19 (Fig 7B, 7C and 7D), although we were only able to reduce MLCK levels by ~45% (Fig 5), (iii) the MLCK inhibitors ML7 and wortmannin also had no effect on LC_20_ phosphorylation at T18 or S19 (Fig 8), and (iv) removal of extracellular Ca^2+^ and depletion of endogenous Ca^2+^ stores with EGTA had no effect on serum-induced LC_20_ phosphorylation at T18 or S19 (Fig 9). MLCK activity is absolutely dependent on Ca^2+^ [14]. We conclude, therefore, that MLCK does not play a role in LC_20_ phosphorylation at T18 or S19 in response to serum treatment of CASMC.

We also investigated the time courses of serum-induced phosphorylation of signaling pathway components distinct from those involved in LC_20_ phosphorylation and its regulation, specifically Akt (S1 Fig), ERK1/2 (S2 and S3 Figs), p38 MAPK (S4A and S4B Fig) and HSP27 (S4C and S4D Fig). Phosphorylation of these proteins at activating sites was much slower than observed for LC_20_, MYPT1 and Par-4 in both CASMC and UASMC.

ROCK knockdown (by 73–77%: Fig 6) reduced the serum-induced diphosphorylation of LC_20_ at T18 and S19 and the phosphorylation of MYPT1 at T696 and T853 and of Par-4 at T163 (Fig 11). ROCK inhibition also reduced LC_20_ diphosphorylation, Par-4 phosphorylation at T163 and MYPT1 phosphorylation at T853 but, unlike the effect of ROCK knockdown, ROCK inhibition did not affect serum-induced phosphorylation of MYPT1 at T696 (Figs 12A–12D and 13A and 13B). This suggests that ROCK knockdown down-regulates a distinct kinase that is responsible for the phosphorylation of MYPT1 at T696 whose activity is unaffected by GSK429286A. Indeed, we have shown previously that ROCK knockdown affects the expression of numerous cellular proteins [33]. ZIPK knockdown (by 45–50%) had a lesser, albeit significant effect on LC_20_ diphosphorylation but actually *increased* MYPT1 and Par-4 phosphorylation (Fig 11A, 11B and 11D). The latter observation can be explained by an increase in ROCK levels due to ZIPK knockdown (Fig 6C). Depletion of ZIPK may also alleviate Par-4-mediated “lockdown” of MYPT1 inhibitory phosphorylation sites, thereby allowing for increased access to ROCK. ZIPK inhibition reduced the serum-induced increase in LC_20_ diphosphorylation but had no effect on MYPT1 or Par-4 phosphorylation (Figs 12A, 12E and 13A and 13C).

As noted above, ZIPK activity is regulated by phosphorylation at multiple sites. Phos-tag SDS-PAGE is a very useful electrophoretic technique that separates phosphorylated forms of a protein based on the interactions of the phosphate moieties with an immobilized ligand in an SDS-polyacrylamide gel such that the higher the phosphorylation stoichiometry the slower the migration rate [35]. We have previously adapted this technique to the analysis of various phosphoproteins, e.g., MYPT1 [84], and in the current work to the phosphorylation of ZIPK. Using Phos-tag SDS-PAGE, we demonstrated that serum treatment of CASMC and UASMC induced a rapid increase in ZIPK phosphorylation at up to four sites (Fig 14). ROCK knockdown inhibited serum-induced ZIPK phosphorylation (Fig 15A), confirming ROCK-mediated phosphorylation of ZIPK originally identified by Haystead’s group [78], while ZIPK knockdown reduced ZIPK levels but did not have a significant effect on ZIPK phosphorylation in response to serum treatment (Fig 15A). Inhibitors of ROCK (GSK429286A) and ZIPK (HS38) individually reduced the serum-induced phosphorylation of ZIPK, with ROCK inhibition having a larger effect (Fig 15B and 15C). ROCK and ZIPK inhibitors together abolished serum-induced ZIPK phosphorylation (Fig 15D). We conclude that ROCK is the predominant kinase responsible for ZIPK phosphorylation in response to serum treatment with additional contribution from ZIPK autophosphorylation.

We used the same strategy combining kinase knockdown and pharmacological inhibitors to investigate the potential roles of MLCK, ROCK and ZIPK in LC_20_ phosphorylation under serum-free conditions (Figs 16 and 17) and concluded that none of these kinases could account for the basal level of LC_20_ phosphorylation that is observed in the absence of serum stimulation.

**Fig 18:**
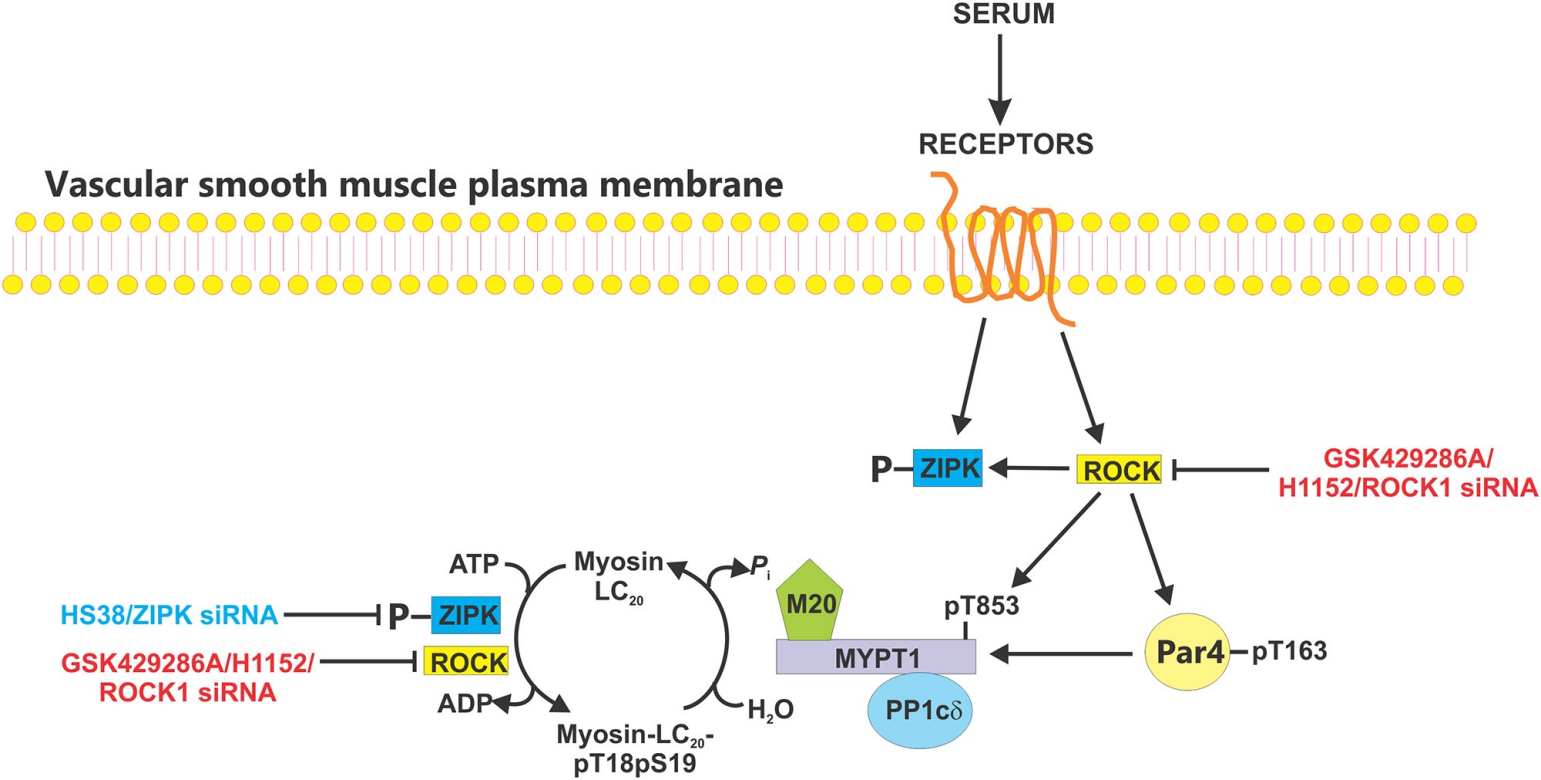
Summary of key findings. A schematic showing the main conclusions from this study regarding serum-induced phosphorylation of the regulatory light chains of myosin II (LC_20_) and the regulatory kinases and phosphatase involved. Serum treatment of CASMC and UASMC activates Rho-associated coiled-coil kinase (ROCK) and zipper-interacting protein kinase (ZIPK) leading to phosphorylation of the regulatory myosin-targeting subunit (MYPT1) of myosin light chain phosphatase (MLCP) at T853 and prostate apoptosis response 4 (Par4) at T163, both of which result in inhibition of MLCP activity. ZIPK activation involves phosphorylation by ROCK and autophosphorylation. Activated ROCK and ZIPK directly phosphorylate LC_20_ at T18 and S19.

Our principal conclusion from these studies is that ROCK plays a major role in serum-induced phosphorylation of LC_20_ at T18 and S19, phosphorylation of MYPT1 at T853 and of Par-4 at T163, whereas ZIPK plays a lesser role in serum-induced diphosphorylation of LC_20_ and does not phosphorylate MYPT1 or Par-4 (Fig 18). Finally, our results indicate the importance of considering ZIPK and ROCK as integrated signaling molecules when interpreting the results of experiments that use genetic knockdown and/or pharmacological inhibition of the kinases.

## Supporting information

S1 Fig Time courses of serum-induced Akt phosphorylation at S473 in human arterial smooth muscle cells. Human coronary (A) and umbilical arterial smooth muscle cells (B) were treated with serum at time zero, as described in the legend to Fig 1. Cells were lysed in Laemmli sample buffer at the indicated times and subjected to SDS-PAGE and western blotting with anti-pS473-Akt. Representative western blots are shown above cumulative quantitative data in each panel. Phospho-Akt signals were normalized to GAPDH and expressed relative to the pAkt: GAPDH ratio at time zero. Values indicate the mean ± SEM (*n* = 12 (A); *n* = 9 (B)). Significant differences from the value at time zero are indicated with their respective *p* values (Dunnett’s *post hoc* test).
(PDF)

S2 Fig Time courses of serum-induced ERK phosphorylation at T202/Y204 (ERK1) or T185/Y187 (ERK2) in human coronary arterial smooth muscle cells. Human coronary arterial smooth muscle cells were treated with serum at time zero, as described in the legend to Fig 1. Cells were lysed in Laemmli sample buffer at the indicated times and subjected to SDS-PAGE and western blotting with anti-pT202/pY204 (ERK1)/anti-pT185/pY187 (ERK2). Representative western blots are shown (A) with cumulative quantitative data for pERK1 (B) and pERK2 (C). Phospho-ERK signals were normalized to GAPDH and expressed relative to the pERK: GAPDH ratio at time zero. Values indicate the mean ± SEM (*n* = 8). Significant differences from the value at time zero are indicated with the actual *p* value or *p < 0.0001 (Dunnett’s *post hoc* test).
(PDF)

S3 Fig Time course of serum-induced ERK1/2 phosphorylation at T202/Y204 and T185/Y187 in human umbilical arterial smooth muscle cells. Human umbilical arterial smooth muscle cells were treated with serum at time zero, as described in the legend to Fig 1. Cells were lysed in Laemmli sample buffer at the indicated times and subjected to SDS-PAGE and western blotting with anti-pT202/pY204 (ERK1)/anti-pT185/pY187 (ERK2). Representative western blots are shown above cumulative quantitative data. Phospho-ERK signals were normalized to GAPDH and expressed relative to the pERK: GAPDH ratio at time zero. Values indicate the mean ± SEM (*n* = 9). Significant differences from the value at time zero are indicated with their respective *p* values (Dunnett’s *post hoc* test).
(PDF)

S4 Fig Time courses of serum-induced p38 MAP kinase phosphorylation at T180 and Y182 and HSP27 phosphorylation at S82 in human arterial smooth muscle cells. Human coronary (A, C) and umbilical arterial smooth muscle cells (B, D) were treated with serum at time zero, as described in the legend to Fig 1. Cells were lysed in Laemmli sample buffer at the indicated times and subjected to SDS-PAGE and western blotting with anti-pT180/pY182-p38 MAP kinase (A, B) or anti-pS82-HSP27 (C, D). Representative western blots are shown above cumulative quantitative data in each panel. Phospho-p38 MAP kinase signals were normalized to SM22 and expressed relative to the phospho-p38 MAP kinase: SM22 ratio at time zero (A, B). Phospho-HSP27 signals were normalized to GAPDH and expressed relative to the pHSP27: GAPDH ratio at time zero. Values indicate the mean ± SEM (*n* = 7). Statistically significant differences from the value at time zero are indicated with their respective *p* values (Dunnett’s *post hoc* test). No statistically significant differences were detected in panel D.
(PDF)

S5 Fig Verification of wortmannin inhibition of Akt phosphorylation. CASMC were serum starved overnight in the presence of H1152 (1 μM), GSK429286A (GSK; 1 μM), wortmannin (1 μM) or vehicle (control). Cells were lysed in Laemmli sample buffer for SDS-PAGE and western blotting with anti-pS473-Akt. Representative western blots are shown in panel A with cumulative quantitative data in panel B. Statistical analysis was carried out with Dunnett’s *post hoc* test.
(PDF)
