# Supplementary figures and images for: Rho-associated kinase and zipper-interacting protein kinase, but not myosin light chain kinase, are involved in the regulation of myosin phosphorylation in serum-stimulated human arterial smooth muscle cells
Source: PLoS One. 2019 Dec 13;14(12):e0226406. doi: 10.1371/journal.pone.0226406 (PMC6910671; doi:10.1371/journal.pone.0226406)

**A**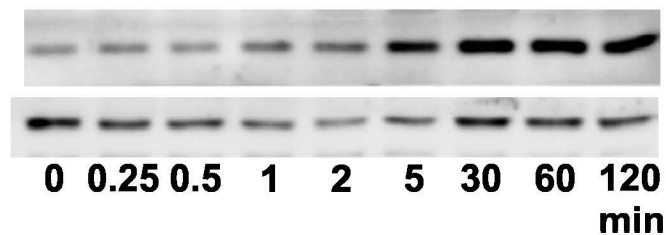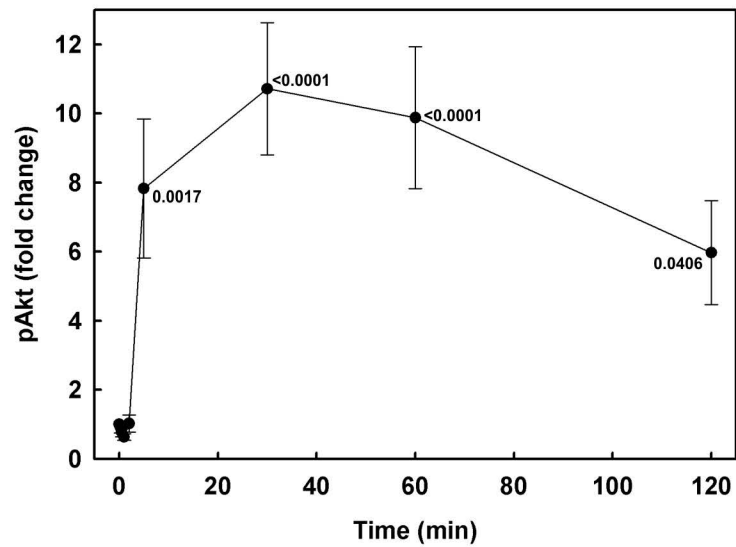**B**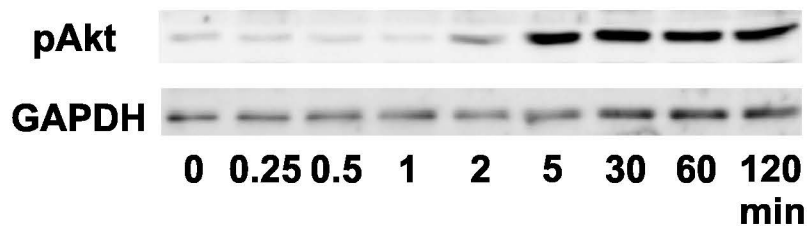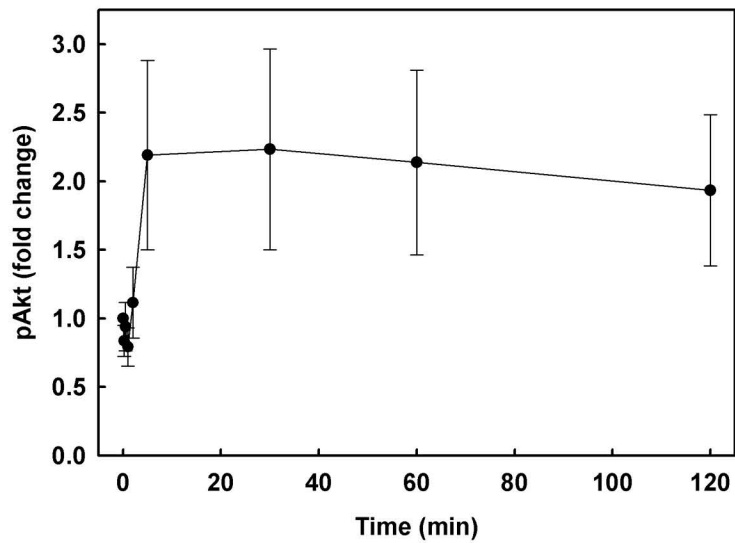

Supplement: S1 Fig — Human coronary (A) and umbilical arterial smooth muscle cells (B) were treated with serum at time zero, as described in the legend to Fig 1. Cells were lysed in Laemmli sample buffer at the indicated times and subjected to SDS-PAGE and western blotting with anti-pS473-Akt. Representative western blots are shown above cumulative quantitative data in each panel. Phospho-Akt signals were normalized to GAPDH and expressed relative to the pAkt: GAPDH ratio at time zero. Values indicate the mean ± SEM (n = 12 (A); n = 9 (B)). Significant differences from the value at time zero are indicated with their respective p values (Dunnett’s post hoc test). (PDF) [file pone.0226406.s001.pdf]

**A**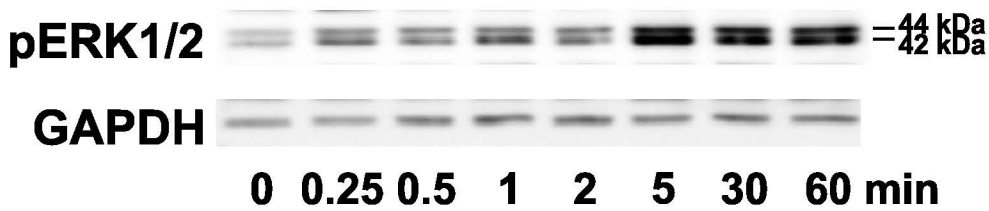**B**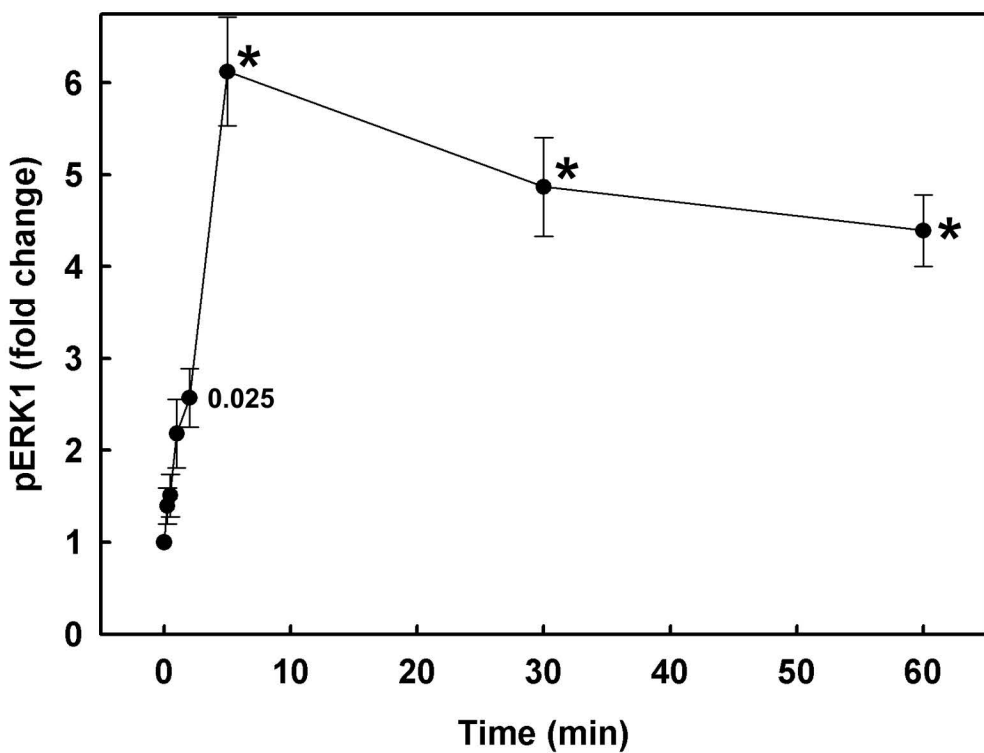**C**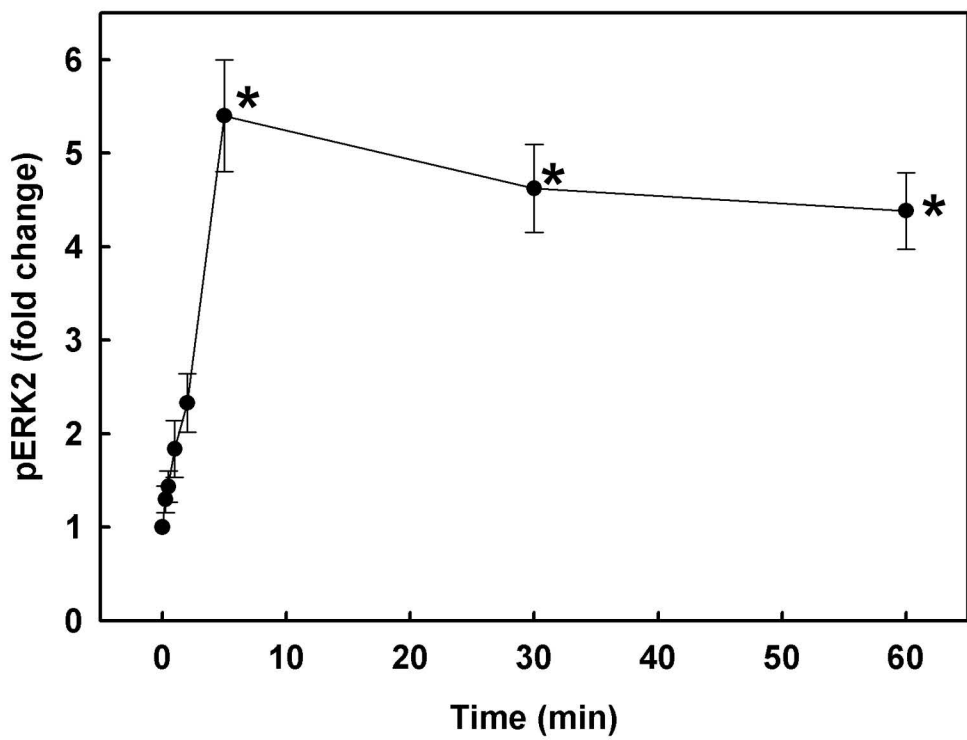

Supplement: S2 Fig — Human coronary arterial smooth muscle cells were treated with serum at time zero, as described in the legend to Fig 1. Cells were lysed in Laemmli sample buffer at the indicated times and subjected to SDS-PAGE and western blotting with anti-pT202/pY204 (ERK1)/anti-pT185/pY187 (ERK2). Representative western blots are shown (A) with cumulative quantitative data for pERK1 (B) and pERK2 (C). Phospho-ERK signals were normalized to GAPDH and expressed relative to the pERK: GAPDH ratio at time zero. Values indicate the mean ± SEM (n = 8). Significant differences from the value at time zero are indicated with the actual p value or *p < 0.0001 (Dunnett’s post hoc test). (PDF) [file pone.0226406.s002.pdf]

**pERK1/2**

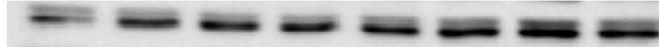

**GAPDH**

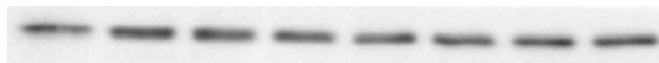

**0 0.25 0.5 1 2 5 30 60 min**

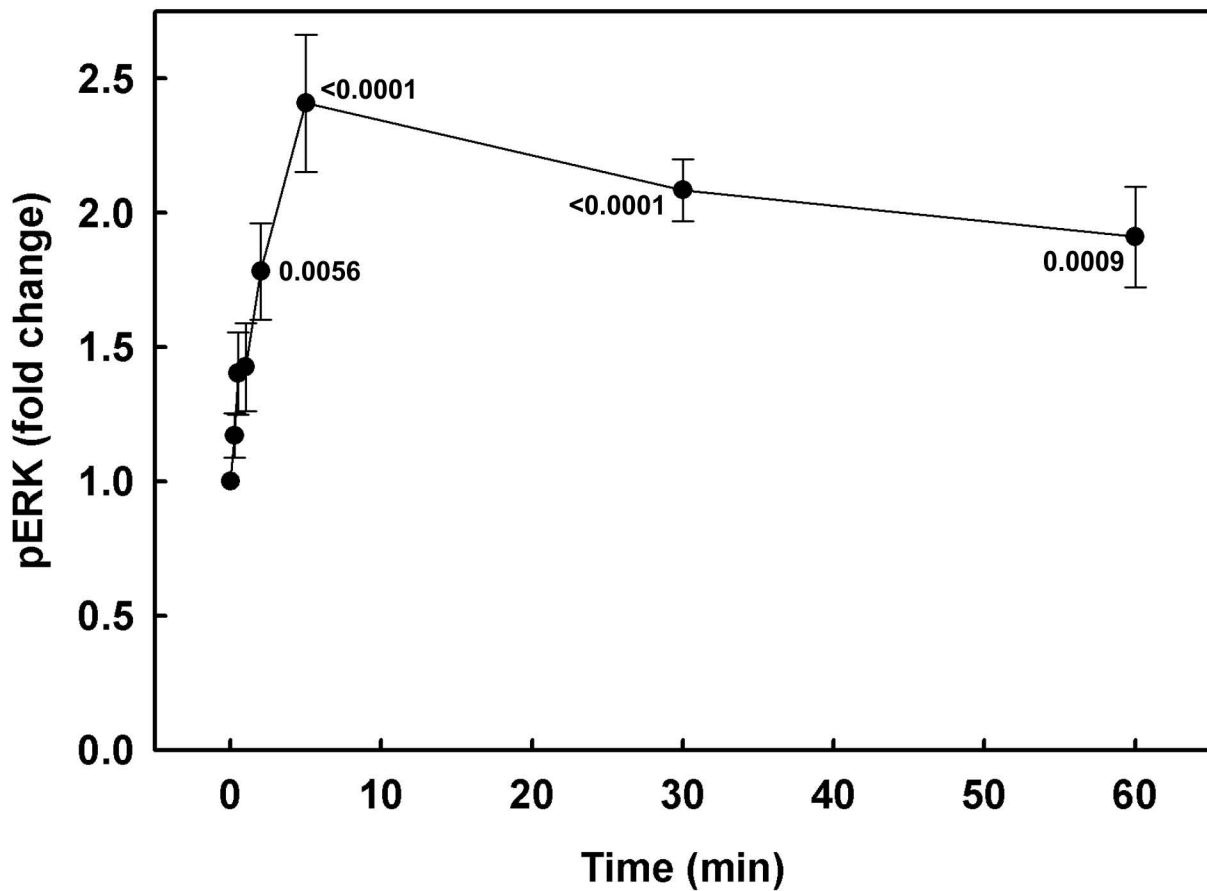

Supplement: S3 Fig — Human umbilical arterial smooth muscle cells were treated with serum at time zero, as described in the legend to Fig 1. Cells were lysed in Laemmli sample buffer at the indicated times and subjected to SDS-PAGE and western blotting with anti-pT202/pY204 (ERK1)/anti-pT185/pY187 (ERK2). Representative western blots are shown above cumulative quantitative data. Phospho-ERK signals were normalized to GAPDH and expressed relative to the pERK: GAPDH ratio at time zero. Values indicate the mean ± SEM (n = 9). Significant differences from the value at time zero are indicated with their respective p values (Dunnett’s post hoc test). (PDF) [file pone.0226406.s003.pdf]

**A**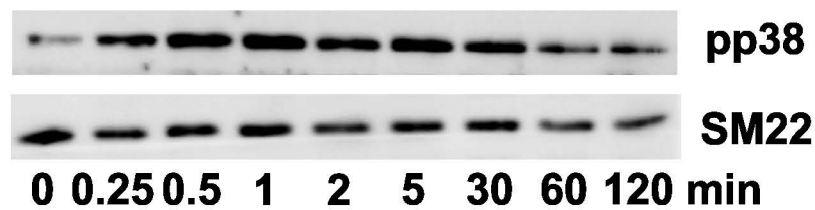

0 0.25 0.5 1 2 5 30 60 120 min

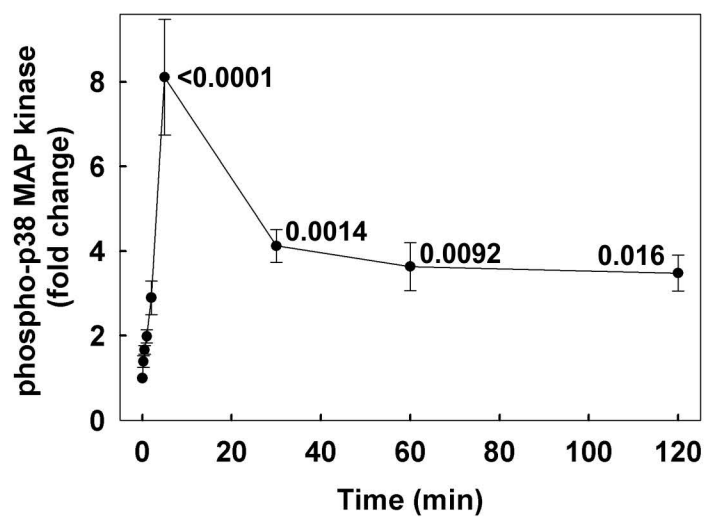**B**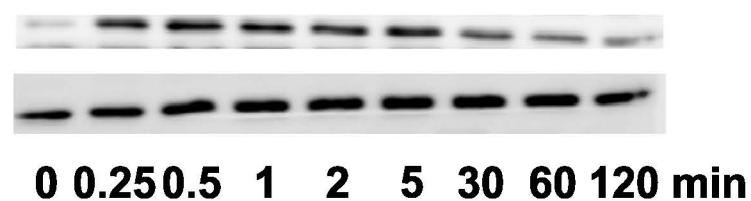

0 0.25 0.5 1 2 5 30 60 120 min

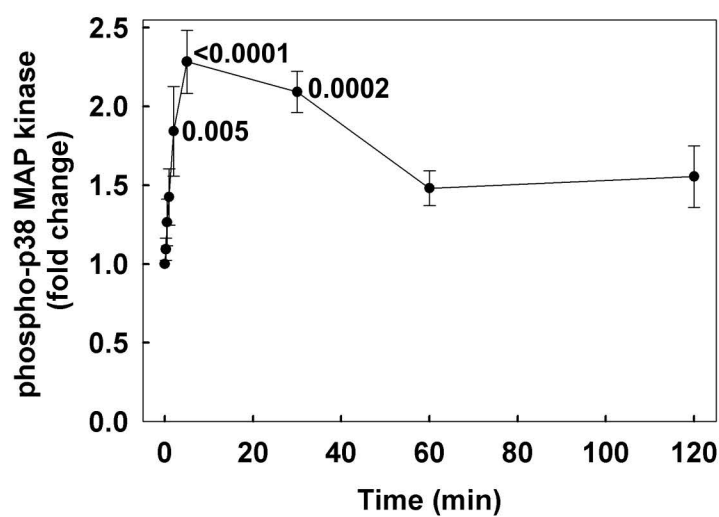**C**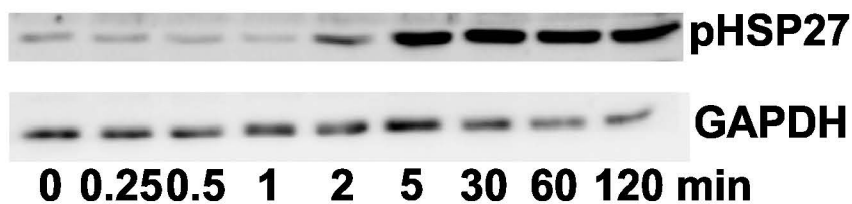

0 0.25 0.5 1 2 5 30 60 120 min

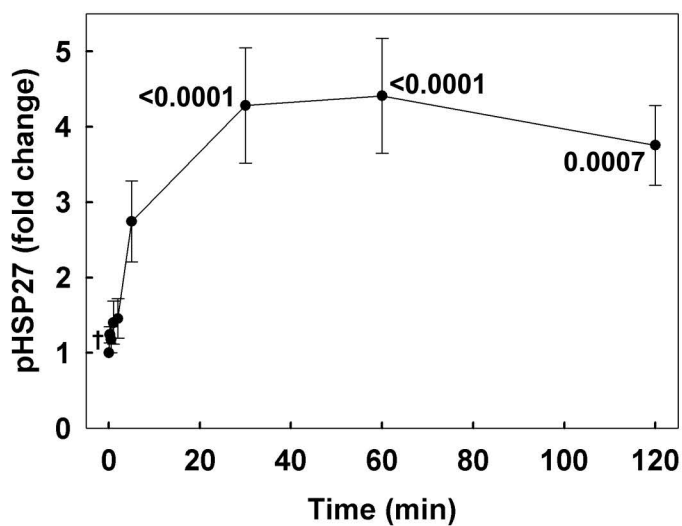**D**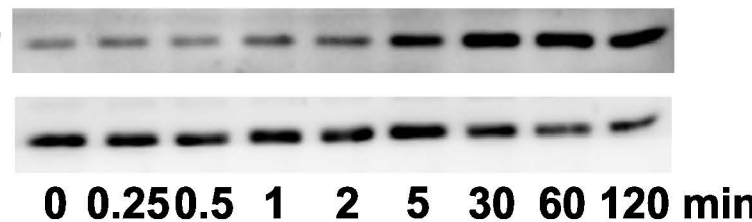

0 0.25 0.5 1 2 5 30 60 120 min

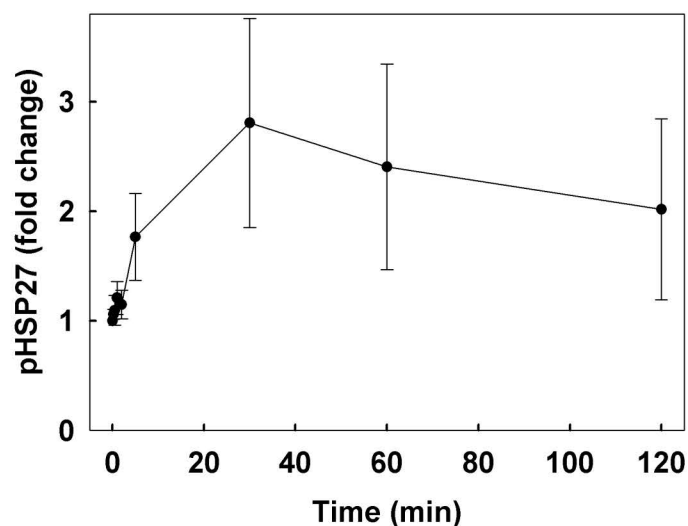

Supplement: S4 Fig — Human coronary (A, C) and umbilical arterial smooth muscle cells (B, D) were treated with serum at time zero, as described in the legend to Fig 1. Cells were lysed in Laemmli sample buffer at the indicated times and subjected to SDS-PAGE and western blotting with anti-pT180/pY182-p38 MAP kinase (A, B) or anti-pS82-HSP27 (C, D). Representative western blots are shown above cumulative quantitative data in each panel. Phospho-p38 MAP kinase signals were normalized to SM22 and expressed relative to the phospho-p38 MAP kinase: SM22 ratio at time zero (A, B). Phospho-HSP27 signals were normalized to GAPDH and expressed relative to the pHSP27: GAPDH ratio at time zero. Values indicate the mean ± SEM (n = 7). Statistically significant differences from the value at time zero are indicated with their respective p values (Dunnett’s post hoc test). No statistically significant differences were detected in panel D. (PDF) [file pone.0226406.s004.pdf]

**A**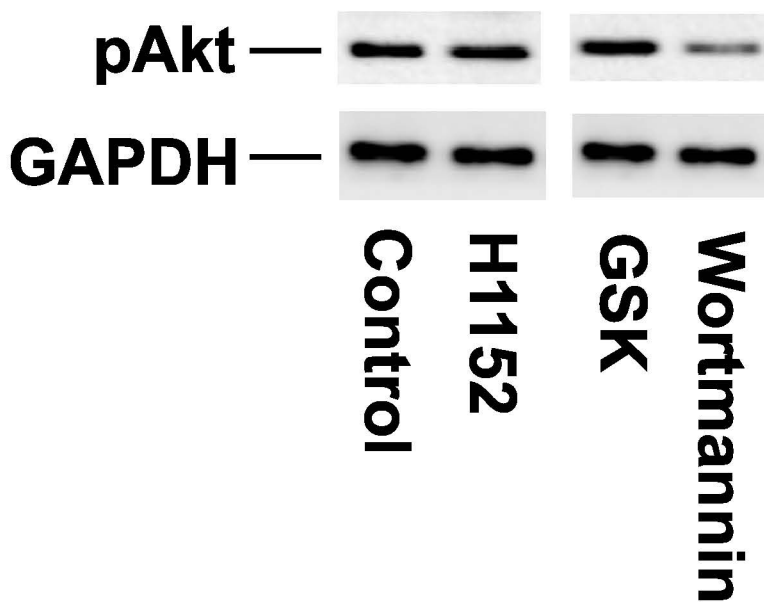**B**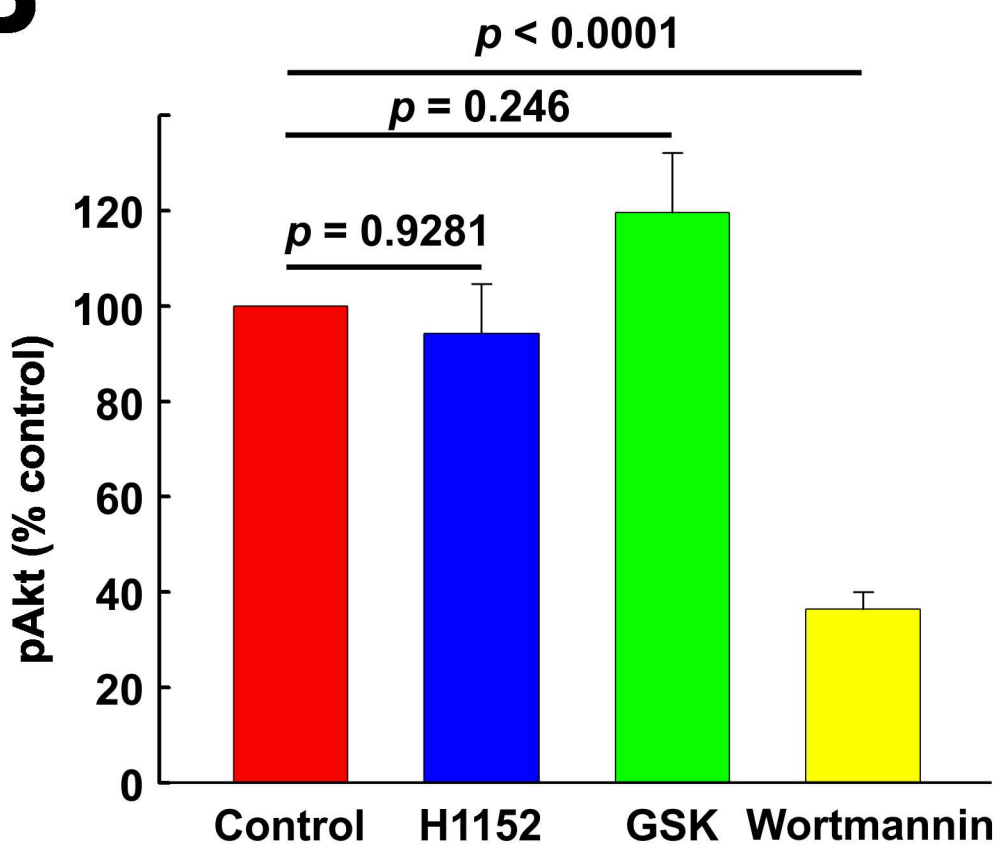

Supplement: S5 Fig — CASMC were serum starved overnight in the presence of H1152 (1 μM), GSK429286A (GSK; 1 μM), wortmannin (1 μM) or vehicle (control). Cells were lysed in Laemmli sample buffer for SDS-PAGE and western blotting with anti-pS473-Akt. Representative western blots are shown in panel A with cumulative quantitative data in panel B. Statistical analysis was carried out with Dunnett’s post hoc test. (PDF) [file pone.0226406.s005.pdf]
